# Whole-Genome Sequencing of 84 Japanese Eels Reveals Evidence against Panmixia and Support for Sympatric Speciation

**DOI:** 10.3390/genes9100474

**Published:** 2018-09-28

**Authors:** Yoji Igarashi, Hong Zhang, Engkong Tan, Masashi Sekino, Kazutoshi Yoshitake, Shigeharu Kinoshita, Susumu Mitsuyama, Tatsuki Yoshinaga, Seinen Chow, Hiroaki Kurogi, Akira Shinoda, Yu-San Han, Ryoshiro Wakiya, Noritaka Mochioka, Toshihiro Yamamoto, Hiroshi Kuwada, Yoshitsugu Kaji, Yutaka Suzuki, Takashi Gojobori, Takanori Kobayashi, Kenji Saitoh, Shugo Watabe, Shuichi Asakawa

**Affiliations:** 1 Department of Aquatic Bioscience, Graduate School of Agricultural and Life Sciences, The University of Tokyo, Bunkyo, Tokyo 113-8657, Japan; aiga@mail.ecc.u-tokyo.ac.jp (Y.I.); schwarze.augen@aliyun.com (H.Z.); tanengkong@gmail.com (E.T.); akyoshita@g.ecc.u-tokyo.ac.jp (K.Y.); akino@mail.ecc.u-tokyo.ac.jp (S.K.); a-mituya@mail.ecc.u-tokyo.ac.jp (S.M.); 2 National Research Institute of Fisheries Science, Japan Fisheries Research and Education Agency, Yokohama, Kanagawa 236-8648, Japan; sekino@affrc.go.jp (M.S.); chow@affrc.go.jp (S.C.); kobayash@fra.affrc.go.jp (T.K.); ksaitoh@affrc.go.jp (K.S.); 3 School of Marine Biosciences, Kitasato University, Sagamihara, Kanagawa 252-0373, Japan; yosinaga@kitasato-u.ac.jp (T.Y.); swatabe@kitasato-u.ac.jp (S.W.); 4 Yokosuka Laboratory, National Research Institute of Aquaculture, Japan Fisheries Research and Education Agency, Yokosuka, Kanagawa 238-0316, Japan; hkuro@affrc.go.jp (H.K.); toshiy@affrc.go.jp (T.Y.); 5 Department of Biology, Tokyo Medical University, Tokyo 160-8402, Japan; shinoda@tokyo-med.ac.jp; 6 Institute of Fishery Science, College of Life Science, National Taiwan University, Taipei 10617, Taiwan; yshan@ntu.edu.tw; 7 Bioresource Sciences, Faculty of Agriculture, Kyushu University, Fukuoka 812-0053, Japan; ryoshiro.wakiya@gmail.com (R.W.); mochioka@agr.kyushu-u.ac.jp (N.M.); 8 Minami-Izu Laboratory, National Research Institute of Aquaculture, Japan Fisheries Research and Education Agency, Kamo, Shizuoka 415-0156, Japan; h.kuwada@yutakanaumi.jp; 9 Wakayama Prefectural Museum of Natural History, Kainan, Wakayama 642-0001, Japan; kaji_y0001@pref.wakayama.lg.jp; 10 Department of Medical Genome Science, Graduate School of Frontier Sciences, University of Tokyo, Kashiwa, Chiba 277-8561, Japan; ysuzuki@k.u-tokyo.ac.jp; 11 Center for Information Biology and DNA Data Bank of Japan, National Institute of Genetics, Mishima, Shizuoka 411-8540, Japan; tgojobor@nig.ac.jp

**Keywords:** genome mapping, genetic patchiness, single nucleotide polymorphisms, population genomics, fixation index, endangered species

## Abstract

The Japanese eel (*Anguilla japonica*), European eel (*Anguilla anguilla*), and American eel (*Anguilla rostrata*) are migratory, catadromous, temperate zone fish sharing several common life cycle features. The population genetics of panmixia in these eel species has already been investigated. Our extensive population genetics analysis was based on 1400 Gb of whole-genome sequence (WGS) data from 84 eels. It demonstrated that a Japanese eel group from the Kuma River differed from other populations of the same species. Even after removing the potential adapted/selected single nucleotide polymorphism (SNP) data, and with very small differences (fixation index [Fst] = 0.01), we obtained results consistently indicating that panmixia does not occur in Japanese eels. The life cycle of the Japanese eel is well-established and the Kuma River is in the center of its habitat. Nevertheless, simple reproductive isolation is not the probable cause of non-panmixia in this species. We propose that the combination of spawning area subdivision, philopatry, and habitat preference/avoidance accounts for the non-panmixia in the Japanese eel population. We named this hypothesis the “reproductive isolation like subset mapping” (RISM) model. This finding may be indicative of the initial stages of sympatric speciation in these eels.

## 1. Introduction

Marine environments contain few physical barriers. The levels of genetic divergence are low in pelagic fishes. Although they inhabit freshwater and brackish water during their long development, the European eel (*Anguilla anguilla*) and American eel (*Anguilla rostrata*) are considered open-ocean fish because their oceanic spawning areas are narrow and their migration routes are long [1,2]. The North Atlantic eels (European and American eels) only spawn in the Sargasso Sea and spend most of their lives in the continental waters ~2000–4000 km (American eel) or 5000–6000 km (European eel) away from their spawning areas [3,4]. It has been argued that they undergo panmixia because their habitats are large and they have single spawning areas [3,4]. Initial studies suggested that panmixia in European eels was unlikely [5]. However, extensive single nucleotide polymorphism (SNP) analyses and population genetics studies demonstrated panmixia in European eels [3,6]. Population studies of American eels indicated that they are also panmictic [7,8]. Based on these recent results, North Atlantic eels have been regarded as a classic example of a panmictic species.

Japanese eels (*Anguilla japonica*) inhabit the northwestern Pacific Ocean, including the waters of northern Philippines, Taiwan, China, Korea, and Japan [1,2]. The spawning area of the Japanese eel was unknown for decades. In the early 2000s, however, it was finally identified as a narrow area near the West Mariana Ridge [9,10]. After hatching out, metamorphosing Japanese glass eels (fry) are dispersed for 2000–3000 km from the spawning area via the warm clockwise currents of the Northwest Pacific Ocean [11]. Like European and American eels, Japanese eels inhabit freshwater and brackish water, and migrate long distances from their single narrow open-ocean spawning areas to inland waters and back to their spawning areas.

Panmixia in Japanese eel has also been studied. To date, several studies only used a few polymorphic markers to investigate panmixia in Japanese eels. Seven of these reported that they failed to determine whether *A. japonica* contains genetically distinct subpopulations [12,13,14,15,16,17,18]. One study, however, reported two non-panmictic groups, indicated by eight polymorphic markers [19]. They divided the Japanese eel population into low-latitude (South China and Taiwan) and high-latitude (Japan, Korea, and Northeast China) groups. The present study attempted to confirm whether panmixia occurs in Japanese eel populations.

We sequenced the individual genomes of 84 Japanese eels collected from Taiwan, Japan, and the West Mariana Ridge. We obtained >1400 Gb (1,400,000 Mb) of sequence data. Therefore, we sequenced data an average of 12-fold per eel. We then identified 30 million SNPs and used them in population genetics analysis. We identified sixty-eight entirely panmictic Japanese eels from Taiwan, Mariana, and four rivers in Japan. We also found a group of adult Japanese eels in the brackish waters of the Kuma River (hereafter, KM individuals), which distinctly differed from the other populations.

Recent population studies of European and American eels have revealed polygenic discrimination of habitat ecotype. There are numerous discriminated SNPs adapted to and/or selected for specific environments or habitats after birth [6,20,21,22]. They are panmictic but harbor many discriminated SNPs suited to specific environments. This phenomenon is called local/habitat/spatially varying selection. We examined whether habitat selections in the brackish water area of the Kuma River could influence non-panmixia in the Japanese eels there. Nevertheless, even after removing all potential selected SNPs from those used in the population genetics analysis, we still confirmed that the Japanese eel population in the Kuma River was non-panmictic. In this report, we show non-panmixia in the Japanese eel of Kuma River, despite their coexistence with the main population. We have compared our findings with the large volume of data on Atlantic eels and will discuss the probable causes and mechanisms of non-panmixis in this Japanese eel subpopulation. Finally, we propose that these findings are indicative of the initial stages of sympatric speciation.

## 2. Materials and Methods

### 2.1. DNA Extraction

**Figure 1:**
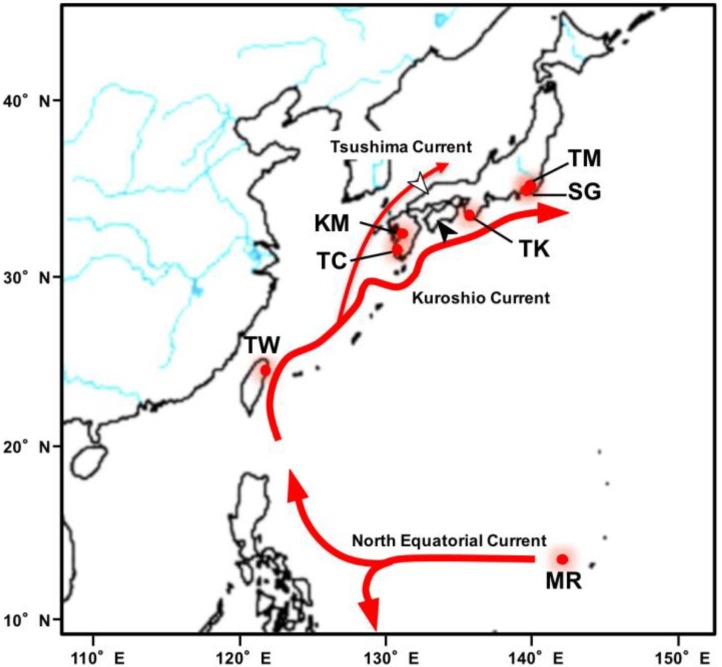
Sampling sites of the Japanese eel (*Anguilla japonica*) used in the present study. SG: Sagami River, Kanagawa Prefecture, Japan; TW: Tainan or Pingtung City, South Taiwan; KM: Kuma River estuary, Kumamoto Prefecture, Japan; TM: Tama River estuary, Tokyo, Japan; TC: Tuchi River, Kagoshima Prefecture, Japan; TK: Takase River, Wakayama Prefecture, Japan; MR: West Mariana Ridge of the Pacific Ocean. White arrowhead: Shimane Prefecture. Black arrowhead: Kochi Prefecture. The eels spawned at MR and the hatched fry were dispersed by ocean currents (red arrows) to coastal areas across East Asia.

Eighty-four samples were used in the analyses. Twenty glass eels were collected from the Sagami River, Kanagawa Prefecture. Another twenty-four were collected in Tainan and Pingtung City, South Taiwan. One yellow to silver eel was collected in the seawater estuary of the Tama River, Tokyo. Sixteen yellow to silver eels were collected in the seawater estuary wetlands of the Kuma River. Thirteen yellow to silver eels were collected in the freshwater part of the Tsuchi River, Kagoshima Prefecture. Three yellow to silver eels were collected in the freshwater part of the Takase River, Wakayama Prefecture. Seven silver eels were collected in the West Mariana Ridge, mid-Pacific Ocean) (Figure 1 and Table S1). Part of the body (mainly muscle) was excised from each eel, immersed in 99.5% *v*/*v* ethanol, and stored at 4 °C until DNA extraction. Genomic DNA was extracted with a DNeasy blood and tissue kit (Qiagen, Hilden, Germany) following the instructions of the manufacturer. The purity of the extracted genomic DNA was assessed by agarose gel electrophoresis. The DNA concentration was determined with a Qubit ds DNA HS assay kit and a Qubit 2.0 fluorometer (Invitrogen, Carlsbad, CA, USA). All animal experiments were approved by the Animal Experiment Committee of the Graduate School of Agricultural and Life Sciences, University of Tokyo (No. P14-952).

### 2.2. Library Preparation and Sequencing

A Nextera DNA sample preparation kit (Illumina, San Diego, CA, USA) was used to prepare the DNA libraries. Indices of the Nextera index kit (Illumina, San Diego, CA, USA) were used for identification purposes. The procedure was followed according to the manufacturer’s instructions. Agencourt Ampure XP beads (Beckman Coulter, Brea, CA, USA) were used to purify tagmented DNA. Library quality, size, and concentration were determined with High Sensitivity D1000 Screen Tape, High Sensitivity D1000 Reagents, and an Agilent 2200 TapeStation (Agilent Technologies, Santa Clara, CA, USA), respectively. DNA library concentrations were determined by qRT-PCR with a KapaLibrary quantification kit (Kapa Biosystems, Wilmington, MA, USA). DNA libraries from the respective samples were mixed at equal concentrations and multiplex-sequenced with a HiSeq 2000 or HiSeq 2500 Sequencer (Illumina, San Diego, CA, USA) to acquire 2 × 100 bp paired-end read sequence data. All samples from each population were randomly mixed by lanes in multiplex sequencing to avoid sequencer or lane bias. Fourteen billion sequencing reads (1400 Gb in total) were deposited in the DNA Data Bank of the Japan Sequence Read Archive (DDBJ SRA) for whole-genome re-sequencing of the Japanese eels (BioProject ID: PRJDB5707).

### 2.3. Analysis Based on Mitochondrial DNA

Mitochondrial DNA (mtDNA) sequence data obtained with a next-generation sequencer were assembled de novo using MIRA4 [23] with reference to the full-length mtDNA sequence of *A. japonica* (accession number AB038556) previously reported [24] for each sample. After alignment with the MEGA6 [25], all sites except the mtDNA protein coding, rRNA, and tRNA regions, were visually eliminated per the methods used for eel and *Tetraodon* pufferfish [26,27]. Molecular phylogenetic trees were reconstructed using 15,649 bases. Nineteen previously reported mtDNA sequences from *Anguilla* species and subspecies were incorporated using the maximum likelihood approach [28]. The common Japanese conger (*Conger myriaster*), Kaup’s arrowtooth eel (*Synaphobranchus kaupii*), and the sawtooth eel (*Serrivomer* sector) were used as an outgroup. The general time reversible (GTR) + gamma distribution (G) + proportion of invariable sites (I) model was used as a molecular evolution model. Bootstrap was performed 1000 times and the data were expressed as percentages.

### 2.4. Mapping

Two “paired-end read” output files were combined per individual for the raw data output. These were used as “single read” data. Low-quality reads were eliminated with Trimmomatic v. 0.33 [29] and FASTX-Toolkit v. 0.0.13 [30]. The respective data for each eel sample were mapped against the *A. japonica* draft genome (GenBank: AVPY00000000.1) [31] with the Burrows-Wheeler Alignment Tool (BWA) version 0.7.12 [32]. Species were also identified with whole-genome sequences (WGS). Three individuals (KM02, KM08, and KM12) from the Kuma River known to be non-panmictic with the main panmictic group were selected (see results). Five individuals (TW22, GS11, TC03, TK02, and MR05) were chosen as representative panmictics. All of these individuals showed read mapping ratios nearly equal to those for the Japanese eel genome sequence. BWA was used to map these eight individuals, with the European eel genome (GenBank: AZBK00000000.1) [33] and the American eel genome (GenBank: ASM160608v1) [34] as reference sequences. Mapping rates were compared. Raw sequence data for the European eel (DDBJ SRA: SRR2046672) and the American eel (DDBJ SRA: SRR5235521) were downloaded and mapped to the genome sequence of each eel. Mapping ratios were then compared.

### 2.5. Single Nucleotide Polymorphism (SNP) Calling and SNP Quality Control

For each sample, the binary alignment map (BAM) file data were sorted and indexed with SAMtools version 1.2 [35]. Duplicated reads were eliminated with the Picard toolkit version 1.138 [36]. The Indel was realigned with Realigner Target Creator and Indel Realigner in the Genome Analysis Toolkit (GATK) version 3.4-46 [37,38]. SNPs were detected in all respective samples with UnifiedGenotyper in GATK.

The total depth of all SNP sites was extracted and a histogram was created with a modal depth of 963. To eliminate SNP sites derived from repeats, SNPs with a total coverage depth in the range of 800–1100 were used in the population structure analysis. Certain SNPs were only observed in one sample and may have contained numerous sequence errors. Therefore, only SNP sites detected in >1 sample (total: 32,312,607) were analyzed.

### 2.6. Genetic Population Analysis Using Single Nucleotide Polymorphism Genotype Data

Scores were assigned to biallelic SNP site genotypes as follows. Sites homozygously different from the reference sequence were designated a value of 2. Sites heterozygously different from the reference sequence were assigned a value of 1. Sites homozygously identical to the reference sequence rated 0. A matrix for 32 million SNP sites was generated for each sample. Cluster and principal component analyses (PCA) were run in the ‘hclust’ function, pvclust packages, and ‘prcomp’ function in R version 3.2.1. with [39,40]. Genetic admixture analysis was performed in STRUCTURE version 2.3.4 [41]. Since the aforementioned matrices were based on the reference sequence, the genetic distance between each sample pair was calculated indirectly. Another scoring matrix was created in which the genetic distance between each pair of samples with bi-allelic heterozygous genotypes was 1 because there were two cases wherein the actual distance was 0 or 2 (Table S2).

### 2.7. Analyses on Each Linkage Group

Single nucleotide polymorphisms on the scaffolds anchored to each chromosome (linkage group) covered ~13% of the eel genome [42]. These were extracted and used in the population analysis. Another newly constructed *A. japonica* genome assembly was also used. In that case, the scaffolds assigned to each chromosome covered ~50% of the genome length [43]. The SNPs assigned to each chromosome were used in cluster analysis, PCA, and genetic admixture analysis.

### 2.8. Fixation Index Values and Tests for Local Selection

Fixation index (Fst) values were calculated for each pair of loci following the methods of Weir and Cockerham [44] and using VCFtools [45] from the variant call format (VCF) file. Localized selection in each chromosome was tested by searching for increases in population differentiation with Fst-based outlier analysis in BayeScan v. 2.1 [46]. BayeScan was run with the default values for all parameters, including a prior odds value of 10, 100,000 iterations, and a burn-in of 50,000 iterations. Loci with a false discovery rate (FDR) of 0.05 were considered for selection. Outliers in 49,585 LG18 SNPs were detected in LOSITAN [47], which was run with 100,000 simulations, a confidence interval of 0.995, and FDR = 0.05.

## 3. Results

### 3.1. Eel Species Identification Based on Full-Length Mitochondrial DNA

The 84 *A. japonica* samples analyzed in the present study originated from seven sites across Japan, Taiwan, and the West Mariana Ridge (Figure 1 and Table S1). The number of reads and the estimated depth (total number of bases/genome size) of the sequence data varied among individuals sampled at each site (Table S3). Molecular phylogenetic trees were developed by incorporating the 19 mtDNA base sequences of all previously reported *Anguilla* species and subspecies. The results showed that the 84 eels analyzed had diverged from the 18 other *Anguilla* species and subspecies and formed a cluster with *A. japonica* AB038556 [24]. Therefore, they were all, in fact, *A. japonica* (Figure S1).

### 3.2. Species Identification Based on the Eel Nuclear Genome

The mtDNA genotype is not always sufficient genetic evidence for species identification. The Japanese eel mtDNA genotypes are often interchangeable with those of the American and European eels [6,48]. Therefore, we evaluated species discrimination reliability by comparing nuclear genomes. As with mtDNA, using a small number of nuclear genes to compare sequences may be misleading. We used BWA to evaluate the overall mapping rate of raw reads for individual eels onto eel genomes [31,33,34]. We chose three non-panmictic individuals (KM02, KM08, and KM12) within the main panmictic groups (shown later) from the Kuma River, and five individuals (TW22, GS11, TC03, TK02, and MR05) from the panmictic groups. The reads of the eight individuals had mapping ratios nearly equal to that for the Japanese eel genome sequence. All individuals had mapping rates ~88%, 84%, and 67% of those for the Japanese, European, and American eel genomes, respectively (Table S4). In contrast, the reads of the European eel had the highest mapping rate to the European eel genome (93.1%) and the American eel had the highest mapping rate to the European eel genome (89.4%). This peculiar result may be explained by the fact that the American eel genome assembly was the least complete of all three species. The reads of the American eel had the highest mapping ratio of all three species to the American eel genome assembly (74.0%) (Table S4). There were no differences between the KM individuals and those from other sample sites. Morphological observation and mitochondrial and nuclear genotype discrimination indicated that these KM individuals were definitely *A. japonica*.

### 3.3. Single Nucleotide Polymorphism Calling

The sequence data with a relatively greater depth of coverage and, therefore, a higher accuracy, were used to distinguish heterozygous and homozygous SNPs and determine the exact genotype. We compared the average read depths on the homozygous and heterozygous sites of each sample and plotted their ratios against the actual depth of coverage of each sample (Figure S2). We aimed for >10-fold the average depth of coverage and successfully obtained it for 75/84 samples (Table S3). We confirmed that depths of 6.9 to 9.7 sufficed to identify each sample by cluster analysis (Figure S3). After BWA mapping, ~128 million SNPs were detected with GATK.

### 3.4. Genetic Population Analysis Using Single Nucleotide Polymorphism Genotype Data for the Whole Genome

**Figure 2:**
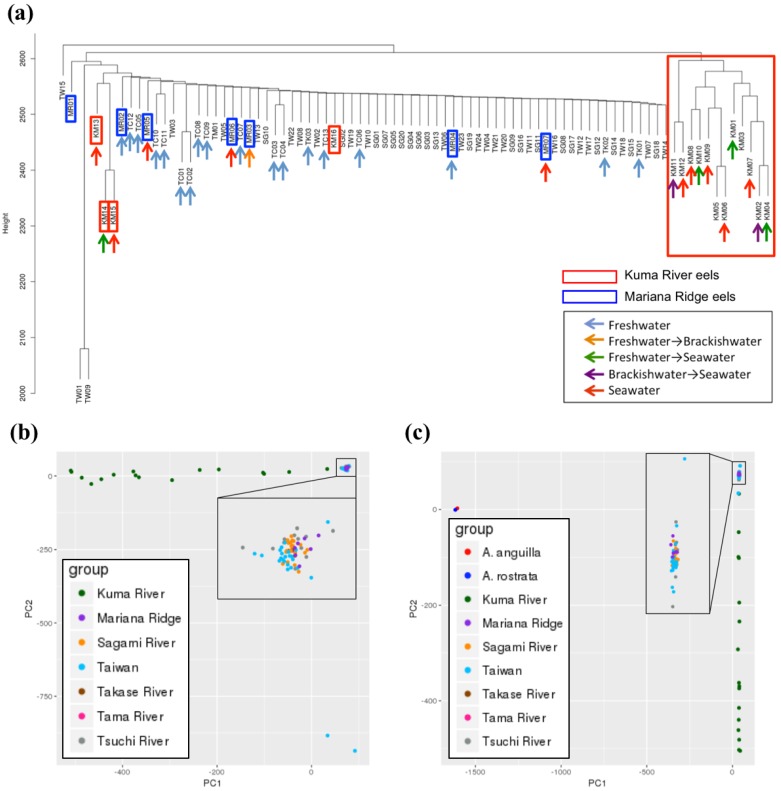
Genetic population analysis of 84 Japanese eel (*Anguilla japonica*) individuals based on 32,312,607 single nucleotide polymorphism (SNP) sites. (**a**) Cluster analysis showed that every sample except KM01–KM12 formed a larger cluster, whereas the 12 KM samples formed a separate smaller cluster; (**b**) Principal component analyses (PCA) of the samples collected in various areas. The Kuma River samples (KM01–KM16) were scattered along the horizontal axis PC1. Therefore, they are distinct from the other populations; (**c**) PCA with the European and American eels. The Japanese eel and the other eels (European eel: *A. anguilla*; American eel: *A. rostrata*) were segregated on the PC1 axis. The Kuma River samples were scattered along the horizontal axis PC2.

The mode of total read depth of coverage was 963 for the 84 samples on each SNP site. We selected the SNPs found in >1 sample and having a total depth of read between 800 and 1100. A total of 32,312,607 SNPs were extracted. They had a calling rate of 99.6%. We genotyped these SNP sites for subsequent analyses. Cluster analysis showed that 72 samples formed one large cluster (Figure 2a), whereas the other 12 samples (KM1-KM12) diverged and formed a separate cluster. The latter were derived from eels collected only in the estuary wetlands of the Kuma River. Therefore, most of the samples collected from Japan (except the Kuma River), Taiwan, and the West Mariana Ridge were panmictic, whereas most of the KM individuals differed from the others.

The principal component analysis (PCA) generated similar results. It plotted all samples at the same point except for the KM individuals (including KM13-KM16), which were scattered across the horizontal PC1 axis. Therefore, they were distinct from all other locations (Figure 2b). We also added the European and American eel sequence data to the PCA. The Japanese and other eels were segregated on the PC1 axis, and the differences among the Japanese eels, including those in the KM group, appeared on the PC2 axis (Figure 2c). The result also suggested a very close relationship between the European and American eel

We also performed a cluster analysis and PCA on several subsets of the ~32 million SNPs and obtained essentially the same results as above (Figures S4 and S5). We tested the cluster analysis with an alternative score matrix (Table S2) for certain samples (Figure S6 and Table S5) and obtained similar results. Therefore, we used the regular matrix in the analysis.

### 3.5. Genetic Population Analysis Using Single Nucleotide Polymorphisms Genotype Data for Each Linkage Group

Single nucleotide polymorphisms with total depths of coverage ranging from 900 to 1000 were investigated for each chromosome to verify whether the KM individuals differed from the other samples for specific chromosomes or the whole genome. Since the reference genome scaffolds were not arranged on chromosomes, in 2014, Kai et al., assigned them to linkage groups (LG) [42]. We extracted SNPs on the scaffolds assigned to linkage groups. The SNPs ranged from 13,825 for LG17 (minimum) to 161,375 for LG9 (maximum) (Table S6). Cluster analysis and PCA for the SNPs in each linkage group provided similar results (Figure S7). We also constructed and used another draft sequence of the Japanese eel as a reference and obtained similar results (Figure S8).

**Figure 3:**
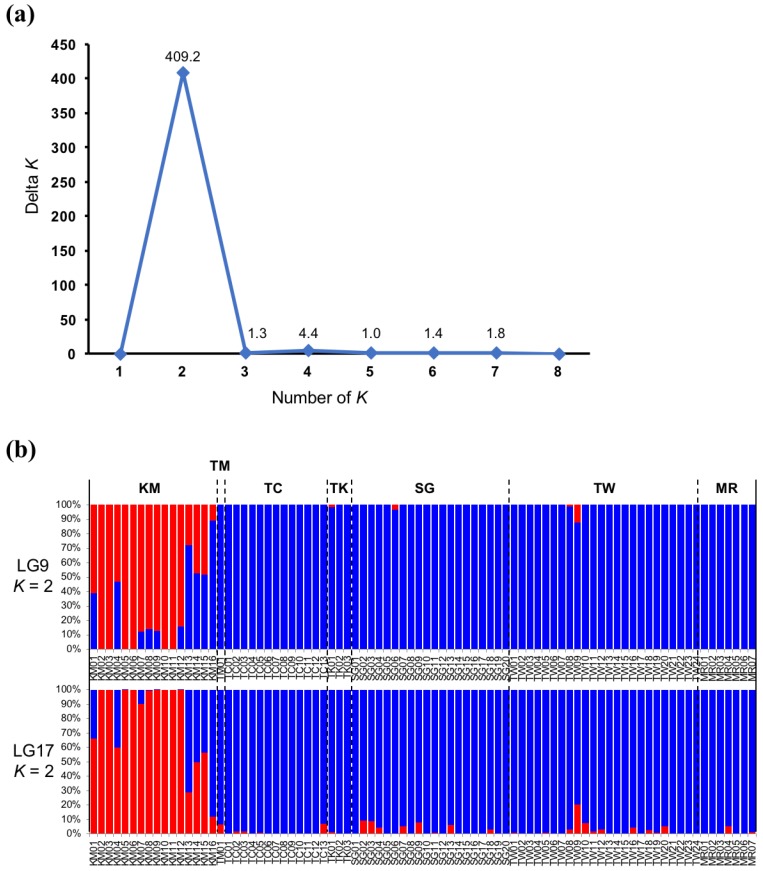
Elucidation of the Japanese eel (*Anguilla japonica*) population structure in STRUCTURE [41]. (**a**) The linkage group (LG) 17 (13,825 SNPs) dataset showed a single peak at *K* = 2. Therefore, these individuals are from two genetically distinct populations; (**b**) Population structure of the Japanese eel based on the SNP sites at LG9 and LG17. Each vertical bar color (red and blue) represents an ancestral population. *K* = 2 was used for analysis; *K* is the number of populations assumed.

We performed genetic admixture analysis in STRUCTURE based on the SNPs for each linkage group. At *K* = 2, the KM individuals were distinct from those of most other localities, with a few exceptions. Therefore, there was a relatively high degree of genetic differentiation between the KM individuals and the other populations (Figure 3 and Figure S9).

Outlier SNP markers indicating natural selection on each chromosome were detected in BayeScan and LOSITAN. Zero to seven outlier SNP sites were detected on each chromosome in BayeScan. In LOSITAN, eight LGs were analyzed and 399–2335 outlier SNP sites were detected (Table S6). Even when these outlier SNP sites were excluded from the cluster analysis and PCA, the Japanese eel of the Kuma River were still segregated from the other populations (Figures S10–S12).

Most of the SNPs characteristic of the KM individuals were commonly distributed across each chromosome. Therefore, these SNPs were not linked to any specific polymorphisms/variants derived from a posteriori environmental selection.

### 3.6. Fixation Index Values for Pairwise Genetic Differentiation

**Figure 4:**
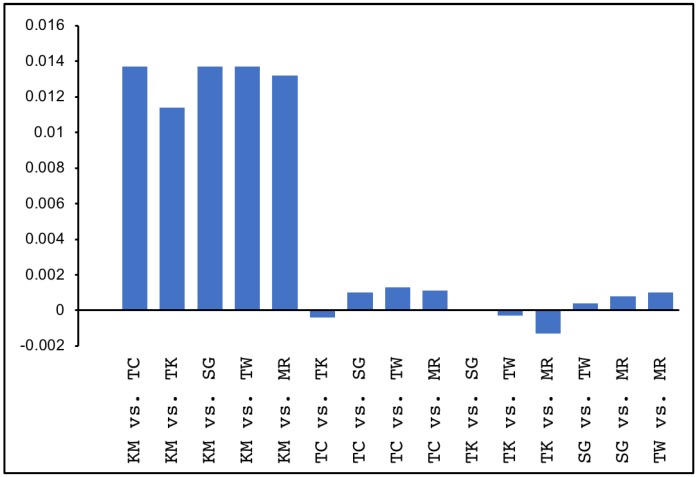
Fixation index (Fst) values between sets of regional Japanese eel (*Anguilla japonica*) populations. The Tama River (TM) individual was removed from the analysis because it consisted of only one sample. Fixation index values were calculated in VCFtools according to the methods of Weir and Cockerham [44].

The fixation index (Fst) values for the 32,312,607 SNP sites were calculated for each locality pair from the VCF file. The average Fst for all SNP sites was ~≤0.001 for eels from the Sagami River (Japan), Taiwan, Tsuchi River (Japan), Takase River (Japan), and West Mariana Ridge (mid-Pacific Ocean). The average Fst was 0.01 between the KM individuals and all other populations (Figure 4 and Table S7). Therefore, there was almost no genetic differentiation among eels from the four rivers in Japan, Taiwan, and the West Mariana Ridge. These groups were almost genetically identical. In contrast, the Fst between the KM individuals and the other groups was ~10-fold greater than those between the other groups. This finding supports the hypothesis that the KM individuals are genetically distinct from the main group.

## 4. Discussion

To identify *Anguilla* species, we examined their nuclear genomes by scoring the raw read mapping rates onto every published eel genome [31,33,34]. To the best of our knowledge, this approach is novel. It is also comprehensive and most likely attributes species correctly (Table S4). This method should also be applicable in discrimination and genetic purity determinations for many other organisms.

The PCA, cluster, and genetic admixture analyses consistently indicated that, except for the KM individuals, the Japanese eels had high genetic uniformity. Previous studies showed that Japanese eel are panmictic. Those reports and the present study generally support the hypothesis that all Japanese eel spawn near the West Mariana Ridge and their larvae are then dispersed to various regions in East Asia [1,2,9,10,11,12,13,14,15,16,17,18].

One previous study reported genetic differences between northern and southern eel groups [19]. However, our results did not indicate any genetic differences among Japanese eels in Japan (except for the KM individuals) and those in Taiwan. In fact, these two correspond to the northern and southern groups of the previous report. Our results were based on several population genetics analyses. These were performed with large numbers of whole genome SNPs, as well as those on individual chromosomes. For any given chromosome, the results were uniform, which indicates that they were highly reliable (Figure 3, Figures S7 and S9). Therefore, we concluded that, within the samples analyzed in the present study, the eels from Taiwan and Japan were panmictic.

These results resembled those reported for Atlantic, American, and European eels. Atlantic eels may be panmictic. Nevertheless, population genetic structures have been reported for them [3,6,7,8]. Fixation index values among the various regional American and European eel groups were very low (0.0003 and 0.0007, respectively) [6,7]. Atlantic and Japanese eels are ecologically similar. Therefore, the panmixia of the Japanese eel is probably comparable to that of the Atlantic eel.

However, our results also show that the KM individuals are genetically distinct from those sampled in other areas (hereafter referred to as the MGJE or the main group of the Japanese eel) (Figure 2, Figure 3 and Figure 4). Therefore, KM individuals may or may not be panmictic with the MGJE.

If the KM population is, in fact, panmictic with the MGJE, then there are three possible explanations for the population genetics differences between them: chaotic genetic patchiness (CGP) [49,50], sweepstakes reproductive success (SRS) [51], or adaptation and/or selection after birth.

Chaotic genetic patchiness is the result of mating among very few parents and incomplete mixing of their larvae with those from other parents en route to the habitat where they develop and mature. In the present study, however, the ages of the KM individuals varied by ≥4–8 years (Table S1). Therefore, the samples in this study did not share common parents. Chaotic genetic patchiness cannot occur without the sharing of a small number of parental eels. Sweepstakes reproductive success is also unlikely because, despite their age differences, the KM individuals were genetically uniform.

Adaptation and/or selection within single generations have been reported for panmictic European and American eels [20,21,22]. Despite their panmixia, however, a few of their SNPs showed a high degree of genetic differentiation. This observation is consistent with divergent natural phenotype selection and/or individual genotype-dependent habitat choice within a single generation. Selection and/or choice may be caused by adaptation to and/or preference for specific salinities and temperatures [6,52,53]. The Kuma River (KM) individuals were captured from the estuary wetlands of the Kuma River. Its environment may differ from those of the other capture locations. It is possible that SNPs with high levels of genetic differentiation influenced the non-panmixia of KM individuals. In our analyses, we chose only eight chromosomes because the number of SNPs the software could process was limited. We detected outlier SNPs on the chromosomes. The fixation index of the outlier SNPs ranged from 0.02 to 1 (mean = 0.23) and the proportion of outlier SNPs was 2.78% (Table S6). These values were higher than those reported for the European eel (outlier SNP Fst range: 0.04 to 0.12; proportion of outlier SNPs: 1.5%) [6]. However, the latter study used SNP selection criteria differing from ours. We removed all outlier SNPs from the data set and repeated the population genetics analysis. The second set of results were consistent with those obtained before outlier SNP removal (Figure S12). Therefore, the primary cause of non-panmixia in the KM individuals was not local or diversifying selection or genotype-dependent habitat choice.

We considered the possibility that KM eels are not panmictic with MGJE. Nevertheless, this scenario is improbable since the spawning area of the Japanese eel is the West Mariana Ridge, and the Kuma River lies within the habitat range of the Japanese eel. A recent study showed no genetic population heterogeneity between eels from Shimane and Kochi [18]. The Kuma River is located between Shimane and the spawning area (Figure 1). Individual KM16 from the Kuma River sample belonged to the major group in the cluster analysis. Therefore, certain individuals in the Kuma River originated from the MGJE. The wide distribution of KM individuals on the PC1 axis (Figure 2b) suggests that KM and MGJE individuals mixed to various extents. This observation was supported by genetic admixture analysis (Figure 3b). The main group of the Japanese eel type (blue) was observed in >50% of all KM individuals and the KM type (red) was found among certain MGJE individuals. These data are evidence of mating between KM and MGJE individuals. Some KM and MGJE individuals (red and blue, respectively) presented with completely different patterns (Figure 3b). However, when we added the European and American eel data to the PCA, all of the KM and MGJE individuals had nearly the same values on the PC1 axis (Figure 2c). Therefore, KM individuals are, in fact, Japanese eels. Taken together, these results suggest there is probably no other spawning area besides the West Mariana Ridge for the Japanese eel.

The West Mariana Ridge is the only spawning area of the Japanese eel and this species may be non-panmictic. Therefore, we must consider the possibility of reproductive isolation among individuals within the same habitat and spawning area. This phenomenon can occur as a result of isolation by distance (IBD) or isolation by time (IBT). Isolation by distance and isolation by time were investigated to understand the genetic population structures of the European eel [54]. However, IBT may not be associated with genetic population heterogeneity in the Japanese eel since the birth years of the KM individuals vary substantially (Table S1). For this reason, IBD may be the cause of genetic population heterogeneity in the Japanese eel.

Some reports suggest that IBD affects panmixia in European eels [5,55] because their spawning areas in the Sargasso Sea are segregated. Since the European eel has natal philopatry, the subdivisions of its spawning areas create its population genetic structures. A comparison of simulated eel dispersal movements with ocean dynamics and real natural coastal eel population genotyping indicated that there is also cryptic female natal philopatry of the European eel in the Sargasso Sea [56].

The spawning areas of the American, European, and Japanese eels are not narrow points, but rather broad volumes extending several hundred kilometers horizontally [1,2,3,4,10]. The Japanese eel may also have multiple spawning subareas and natal philopatry. These may contribute to the genetic population heterogeneity of this species.

Multiple spawning areas and natal philopatry may not fully explain the genetic population heterogeneity of the Japanese eel. While certain reports showed genetic population structures in the European eel, they were taken to mean relatively less random mixing in their populations (that is, CGP/SRS) [49,50,51] or IBD and IBT [54]). Local selection or polygenic discrimination in single generations may also affect genetic population structures [6,21]. Even if these genetic events affect genetic population structures in the American and European eel, these species are panmictic because these genetic population structures are temporal and unstable.

The attributes of the KM individuals differ from those of European eels. The Kuma River (KM) individuals were adults differing in age by ≥4–8 years. It is, therefore, difficult to explain their genetic population heterogeneity with CGP and SRS (Table S1). In general, CGP is observed in young fry populations [49]. However, one study reported CGP in adult European eels [50]. Fixation index between a group of individuals in the Tiber River in 2002 and other groups (the Lesina River in 2002, the Lisina River in 2004, and the Capolace River in 2000) were as little as 0.011–0.015 of those measured between the KM individuals and the other Japanese eel groups. However, Fst between the Tiber River in 2004 and the other groups were much smaller (0.001–0.004). This wide variance in Fst at the same location indicates that high Fst were temporal rather than regional and were caused by CGP. Although the age of the KM individuals varied widely, all of them were genetically distinct from the MGJE. Therefore, these differences were the effect of regional factors rather than CGP. The wide range of ages among the KM individuals also suggests that IBT probably does not account for the observed genetic differences.

At first glance, the local selection in a single generation observed in the Japanese eel seems to be the most probable cause of the genetic distinction between the KM individuals and the MGJE. The habitat of the KM individuals is estuarine and clearly differs from the open ocean habitat. In addition, strong genetic differences were uniformly distributed on each chromosome. This uniformity resembles the flat Fst distribution around the selected loci of the selected individual panmictic European eels. However, the scores and proportions of outlier SNPs for the KM individuals were considerably higher than those for the European eel (Table S6). A flat Fst distribution around the selected loci is a characteristic of selection within a single generation [6]. However, a flat Fst distribution is also a feature of genome hitchhiking [57,58,59,60]. The apparent structure after the removal of outlier SNPs suggests that most of the differential SNPs between the KM individuals and the MGJE differed from those observed in the Atlantic eels (Figure S12). The differential SNPs of the Japanese eel may have been generated by genome hitchhiking, which causes speciation after many generations. In contrast, the differential SNPs in the Atlantic eels were not inherited by subsequent generations [6].

To establish this model, reproductive isolation is required. Decades of exploring the spawning area of the Japanese eel strongly suggests that this species has no other spawning ground besides the West Mariana Ridge. Consequently, IBD or subdivision within the existing West Mariana Ridge spawning area is probably the best model to explain the comparatively higher genetic difference of KM individuals relative to the MGJE. Moreover, natal philopatry is also an essential factor in the development of KM individuals.

However, other factors in addition to IBD and philopatry may segregate the KM individuals. The estuarine wetland of the Kuma River is located within a larger area inhabited by the Japanese eel. It could, therefore, be traversed by the Japanese glass eel (Figure 1). Nevertheless, in the Kuma River, there are many individuals with the KM genotype, but MGJE eels are relatively rare there. Local selection could also explain the development of the KM Japanese eel subpopulation. Individuals with the KM genotype may prefer the estuarine environment of the Kuma River and/or MGJE eels may avoid it. Salinity preference in eels is associated with habitat selection among other eel behaviors [61].

**Figure 5:**
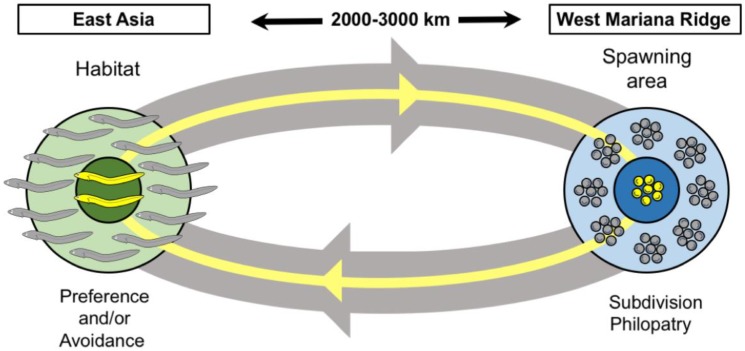
The “reproductive isolation like subset mapping” (RISM) model explaining the generation of the Kuma River Japanese eel population (KMs). Gray eggs and eels indicate the main group of the Japanese eel (MGJE). Yellow eggs and eels indicate those of KM individuals. Thick grey and thin yellow lines indicate the migration routes of the MGJE and KM individuals, respectively. Arrows indicate migration direction. Light green area indicates the MGJE habitat in East Asia. Dark green area shows the estuary Kuma River wetland within the Japanese eel habitat. Light blue area shows the MGJE spawning area in the West Marian Ridge. Dark blue area shows the spawning area subdivision occupied by the KM individuals. Reproductive isolation was achieved by a combination of spawning area subdivision, philopatry, and relative differences in Kuma River environmental (salinity, temperature) preferences between the KM individuals and the MGJE. Consequently, there is a non-panmictic coexistence of the KM individuals and the MGJE, which may be the initial stage of sympatric speciation.

Integrating the aforementioned ideas, we present a hypothesis to explain the generation of KM subpopulations (Figure 5). We named this hypothesis the “reproductive isolation like subset mapping” (RISM) model. The basis of this hypothesis is reproductive isolation explained in part by subdivision of the spawning area and philopatry and in part by the preference of the KM and/or MGJE individuals for different environments (salinity, temperature) within the Kuma River. The combination of these factors results in the non-panmictic coexistence (or near-coexistence) of the KM and MGJE subpopulations. The results of the present study, then, may indicate the initial stages of sympatric specification.

In the aforementioned scenario, there should be a KM-type Japanese glass eel subpopulation. Therefore, identification of these KM individuals is one way to test this hypothesis. Moreover, all spawning area subdivisions must be defined and the habitat preferences of the eels in the Kuma River and elsewhere must be studied.

The spawning areas of the American and European eels overlap in the Southern Sargasso Sea. Certain individuals from both species are known to mix. However, the two species are, and remain, different from each other. Several eel pairs have overlapping habitats and/or spawning areas, but continue to be independent as species. Our hypothesis may explain this sympatry and eel speciation.

Although we performed WGS on 84 eels, in practice, fewer SNPs may have sufficed for us to arrive at the same conclusions stated above. RAD-tagging and other similar or related methods may be useful in further studies of the Japanese eel and other organisms. However, WGS generates the highest density of SNPs data, facilitates rapid and comprehensive identification of the genes associated with specific phenotypes, and detects ancient gene flows and structural polymorphisms. Aided by new genomic data [43], our SNP analysis will be refined. If the cost of sequencing continues to decrease in the future, it will be easier to use WGS to perform population genetics on a large number of individuals.

## 5. Conclusions

Temperate zone eels have several characteristic ecological features, including continent-size habitats and single narrow spawning areas. Despite their large biocycles, panmixia (random mating within a population) has been reported in these species. In the present study, however, we found evidence against panmixia in the Japanese eel. Initially, it was believed that this behavior could be explained by reproductive isolation. Nevertheless, the West Mariana Ridge is the only spawning area of the Japanese eel and reproductive isolation there is highly unlikely. We proposed the RISM model to understand this phenomenon. Finally, from the economic aspect, our results may indicate that various genetic populations can be “branded” independent of their aquaculture area and morphology, and would require the revision of resource management policies.

## Supplementary Materials

The following are available online at http://www.mdpi.com/2073-4425/9/10/474/s1: Figure S1: Molecular phylogenetic tree constructed by the maximum likelihood method on the whole mitochondrial DNA sequences from the 84 Japanese eels *Anguilla japonica* assembled in this study and 19 *Anguilla* species reported previously, Figure S2: Plots of the ratio of average depth of coverage at SNP sites regarded as homozygous and heterozygous against the sequence depth of samples, Figure S3: Cluster analysis using half of the raw data of 9 representative samples together with the 84 samples, Figure S4: Genetic population analyses at 3 different sequence depths for the Japanese eel *Anguilla japonica* collected from different areas, Figure S5: Genetic population analysis was based on 4,557,695 major SNPs that were present in 15 to 66 of the 84 samples, Figure S6: Molecular phylogenetic tree based on calculated pairwise genetic distance, Figure S7: Genetic analysis of the 84 sampled Japanese eels *Anguilla japonica* based on SNP sites at different linkage groups of *A. japonica* ranged from 13,825 for LG17 (minimum) to 161,375 for LG9 (maximum), Figure S8: Cluster analysis of 84 Japanese eel *Anguilla japonica* individuals based on SNP sites extracted using another draft genome sequence of *A. japonica* assembled by ourselves as a reference (genome size: 1.16 Gb, number of scaffolds: 186743), Figure S9: Population structure of the 84 Japanese eels by STRUCTURE software based on the SNP sites of each *Anguilla japonica* linkage group, Figure S10: Detection of outlier SNPs in each linkage group using BayeScan, Figure S11: Detection of outlier SNPs in the 49,585 SNPs of LG18 using LOSITAN, Figure S12: Cluster analysis and PCA of 84 sampled Japanese eels *Anguilla japonica* based on SNP sites at different linkage groups of *A. japonica* without outlier SNP sites, Table S1: Summary of the samples of *A. japonica* used in this study, Table S2: The score matrix to calculate the pairwise distance, Table S3: Summary of sequence data obtained for each sample, Table S4: Comparison of mapping rates with eels of different species, Table S5: Pairwise distance values among 30 individuals of Japanese eel *Anguilla japonica* based on 32,312,607 SNP sites, Table S6: The number of SNPs and the outlier SNPs site in each linkage groups, and Table S7: Fixation index values between each set of six regional populations of the Japanese eel *Anguilla japonica*, based on 32,312,607 SNP sites.
