# Supplementary material for: Whole-Genome Sequencing of 84 Japanese Eels Reveals Evidence against Panmixia and Support for Sympatric Speciation
Source: Genes (Basel). 2018 Sep 28;9(10):474. doi: 10.3390/genes9100474 (PMC6210723; doi:10.3390/genes9100474)
Supplement: Supplementary file 1 [file genes-09-00474-s001.pdf]

genes

MDPI

Supplementary Materials for

Article

Whole-genome sequencing of 84 Japanese eels reveals evidence against panmixia and support for sympatric speciation

Yoji Igarashi [1], Hong Zhang[1], Engkong Tan [1], Masashi Sekino[2], Kazutoshi Yoshitake [1], Shigeharu Kinoshita [1], Susumu Mitsuyama [1], Tatsuki Yoshinaga [3], Seinen Chow [2], Hiroaki Kurogi [4], Akira Shinoda [5], Yu-San Han [6], Ryoshiro Wakiya [7], Noritaka Mochioka [7], Toshihiro Yamamoto [4], Hiroshi Kuwada [8], Yoshitsugu Kaji [9], Yutaka Suzuki [10], Takashi Gojobori [11], Takanori Kobayashi [2], Kenji Saitoh [2], Shugo Watabe [3] and Shuichi Asakawa [1,*]

[1] Department of Aquatic Bioscience, Graduate School of Agricultural and Life Sciences, The University of Tokyo, Bunkyo, Tokyo 113-8657, Japan; aiga@mail.ecc.u-tokyo.ac.jp (Y.I.); schwarze.augen@aliyun.com (H.Z.); tanengkong@gmail.com (E.T.); akyoshita@g.ecc.u-tokyo.ac.jp (K.Y.); akino@mail.ecc.u-tokyo.ac.jp (S.K.); a-mituya@mail.ecc.u-tokyo.ac.jp (S.M.)
[2] National Research Institute of Fisheries Science, Japan Fisheries Research and Education Agency, Yokohama, Kanagawa 236-8648, Japan; sekino@affrc.go.jp (M.S.); chow@affrc.go.jp (S.C.); kobayash@fra.affrc.go.jp (T.K.)
[3] School of Marine Biosciences, Kitasato University, Sagamihara, Kanagawa 252-0373, Japan; yosinaga@kitasato-u.ac.jp (T.Y.); swatabe@kitasato-u.ac.jp (S.W.)
[4] Yokosuka Laboratory, National Research Institute of Aquaculture, Japan Fisheries Research and Education Agency, Yokosuka, Kanagawa 238-0316, Japan; hkuro@affrc.go.jp (H.K.); toshiy@affrc.go.jp (T.Y.)
[5] Department of Biology, Tokyo Medical University, Tokyo 160-8402, Japan; shinoda@tokyo-med.ac.jp
[6] Institute of Fishery Science, College of Life Science, National Taiwan University, Taipei 10617, Taiwan; yshan@ntu.edu.tw
[7] Bioresource Sciences, Faculty of Agriculture, Kyushu University, Fukuoka 812-0053, Japan; ryoshiro.wakiya@gmail.com (R.W.); mochioka@agr.kyushu-u.ac.jp (N.M.)
[8] Minami-Izu Laboratory, National Research Institute of Aquaculture, Japan Fisheries Research and Education Agency, Kamo, Shizuoka 415-0156, Japan
[9] Wakayama Prefectural Museum of Natural History, Kainan, Wakayama 642-0001, Japan; kaji_y0001@pref.wakayama.lg.jp
[10] Department of Medical Genome Science, Graduate School of Frontier Sciences, University of Tokyo, Kashiwa, Chiba 277-8561, Japan; ysuzuki@k.u-tokyo.ac.jp
[11] Center for Information Biology and DNA Data Bank of Japan, National Institute of Genetics, Mishima, Shizuoka 411-8540, Japan; tgojobor@nig.ac.jp
† Present address: National Association for the Promotion of Productive Seas, Kodenmacho Matsumura Bldg, Nihonbashi Kodenmacho, Chuo, Tokyo 103-0001, Japan; h.kuwada@yutakanaumi.jp
‡ Present address: Tohoku National Fisheries Research Institute, Japan Fisheries Research and Education Agency, Shiogama, Miyagi 985-0001, Japan; ksaitoh@affrc.go.jp
* Correspondence: asakawa@mail.ecc.u-tokyo.ac.jp; Tel.: +81-3-5841-5296

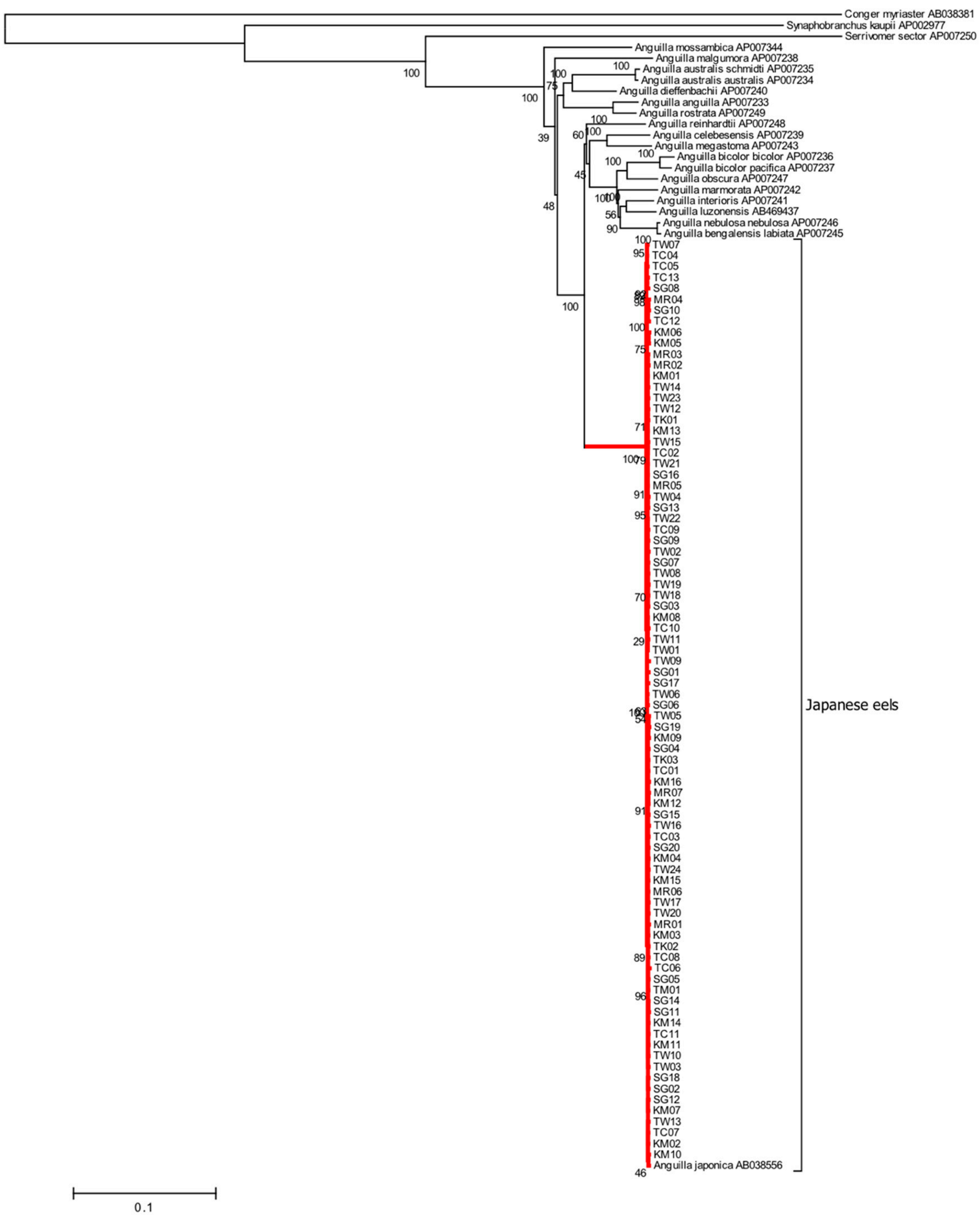

Figure S1. Molecular phylogenetic tree constructed by the maximum likelihood method on the whole mitochondrial DNA sequences from the 84 Japanese eels *Anguilla japonica* assembled in this study and 19 *Anguilla* species reported previously. Common Japanese conger (*Conger myriaster*, AB038381), Kaup's arrowtooth eel (*Synaphobranchus kaupii*, AP002977), and sawtooth eel (*Serrivomer* sector, AP007250) were used as outgroups. The bootstrap probabilities from a 1,000 replicate analyses are given as percentages at the nodes. The results confirmed that all of the 84 eels used in this study are *A. japonica*.

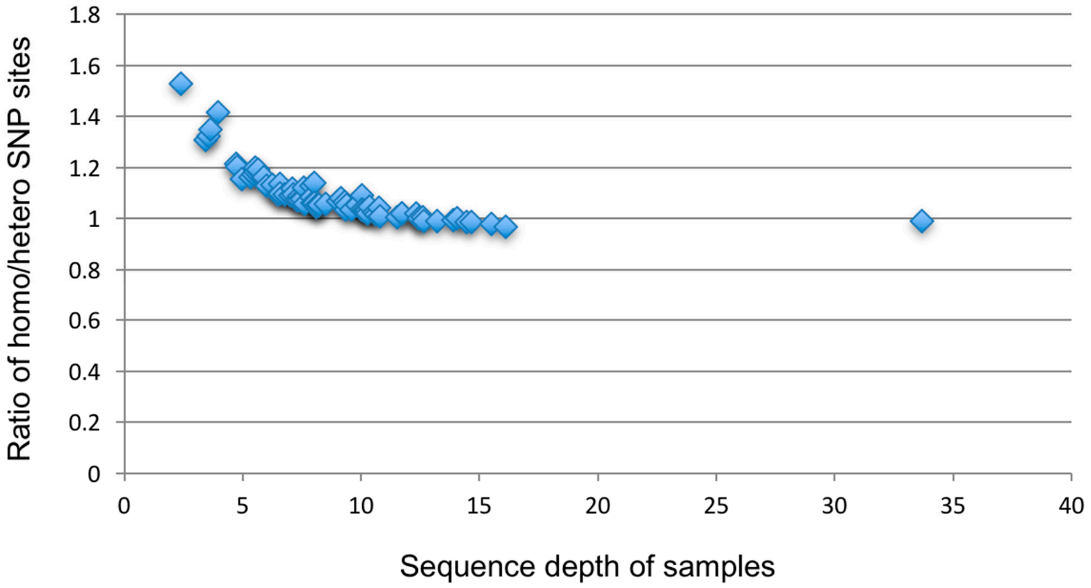

Figure S2. Plots of the ratio of average depth of coverage at SNP sites regarded as homozygous and heterozygous against the sequence depth of samples. To distinguish heterozygous SNPs from homozygous SNPs and determine the genotype exactly, sequences with greater depth are more accurate. If the depth is sufficient and the genotyping is accurate, the ratio is expected to converge to 1.

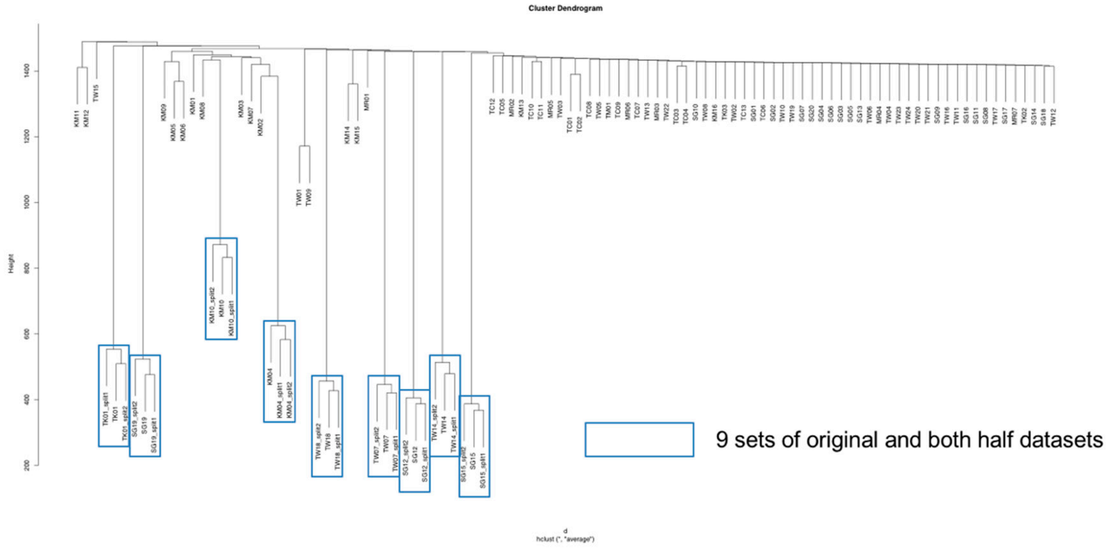

Figure S3. Cluster analysis using half of the raw data of 9 representative samples together with the 84 samples. Each half data of each sample (the depth of coverage are from 6.9 to 9.7) was independently processed. For all of 9 samples, each-half data and original full data formed a cluster with a closer exclusive relationship, indicating that sequences with a depth of 6.9 or more are sufficient.

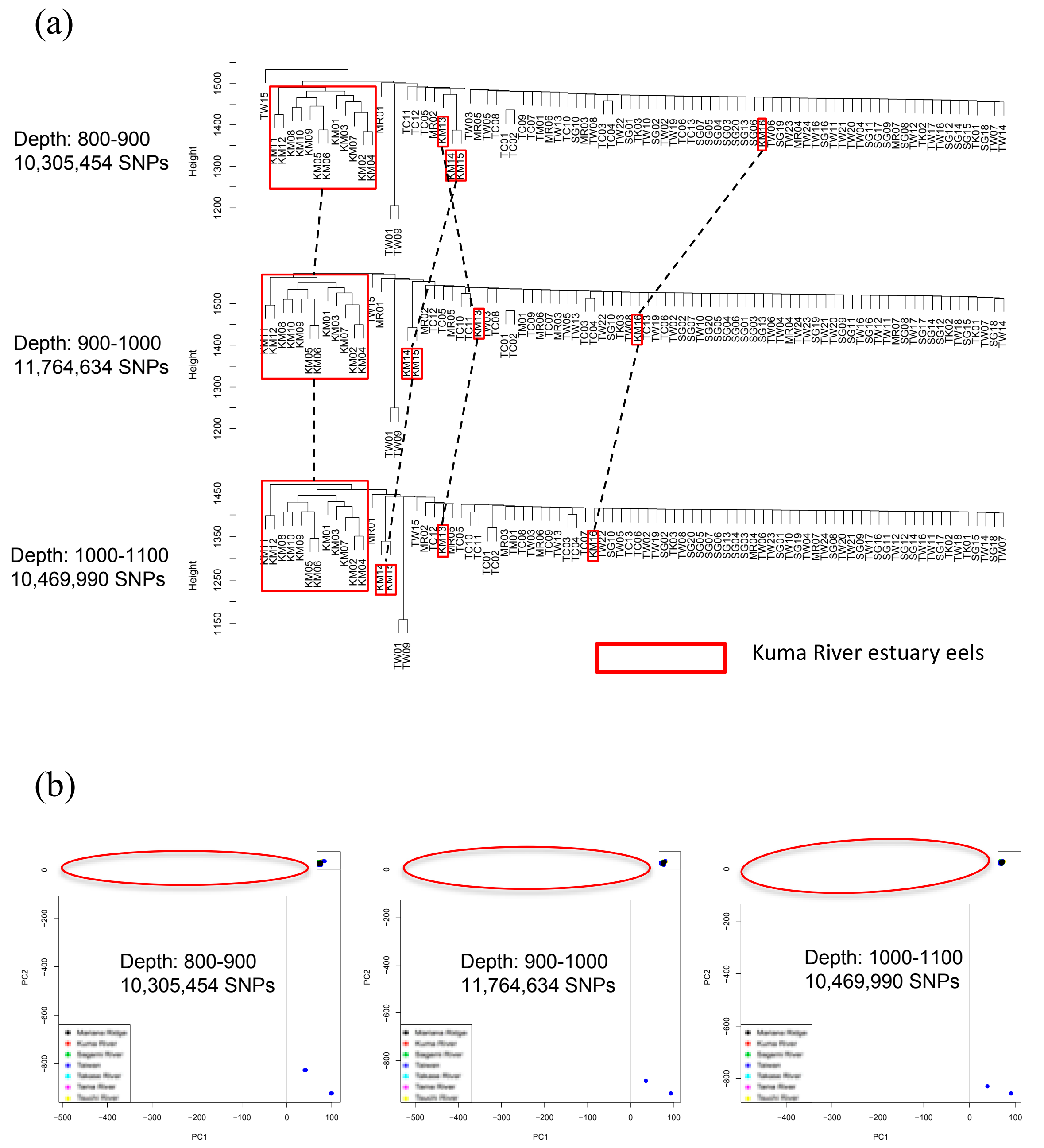

Figure S4. Genetic population analyses at 3 different sequence depths for the Japanese eel *Anguilla japonica* collected from different areas. (**a**) Cluster analysis and (**b**) PCA. The depth ranges of 800 to 900, 900 to 1000, and 1000 to 1100 include 10,305,454, 11,764,634, and 10,469,990 SNP sites, respectively.

(a)

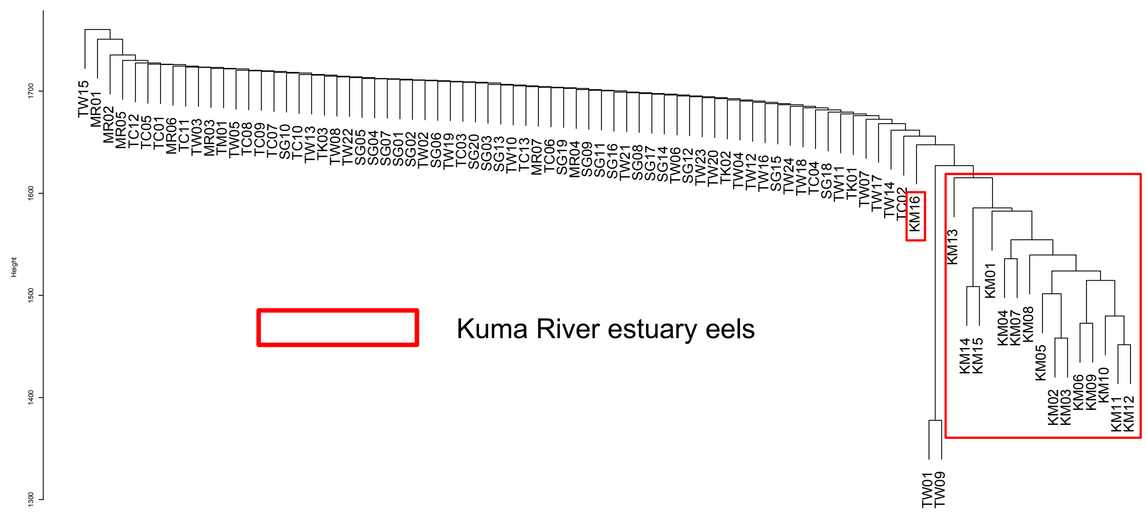

(b)

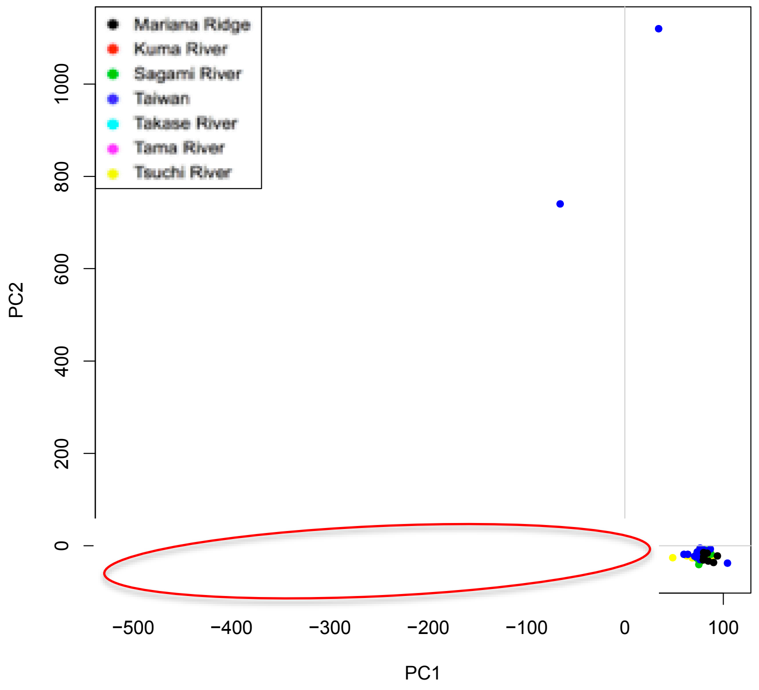

Figure S5. Genetic population analysis was based on 4,557,695 major SNPs that were present in 15 to 66 of the 84 samples. (**a**) Cluster analysis and (**d**) PCA were performed using the SNPs. This result confirmed that it was not just certain minor SNPs characterizing the samples.

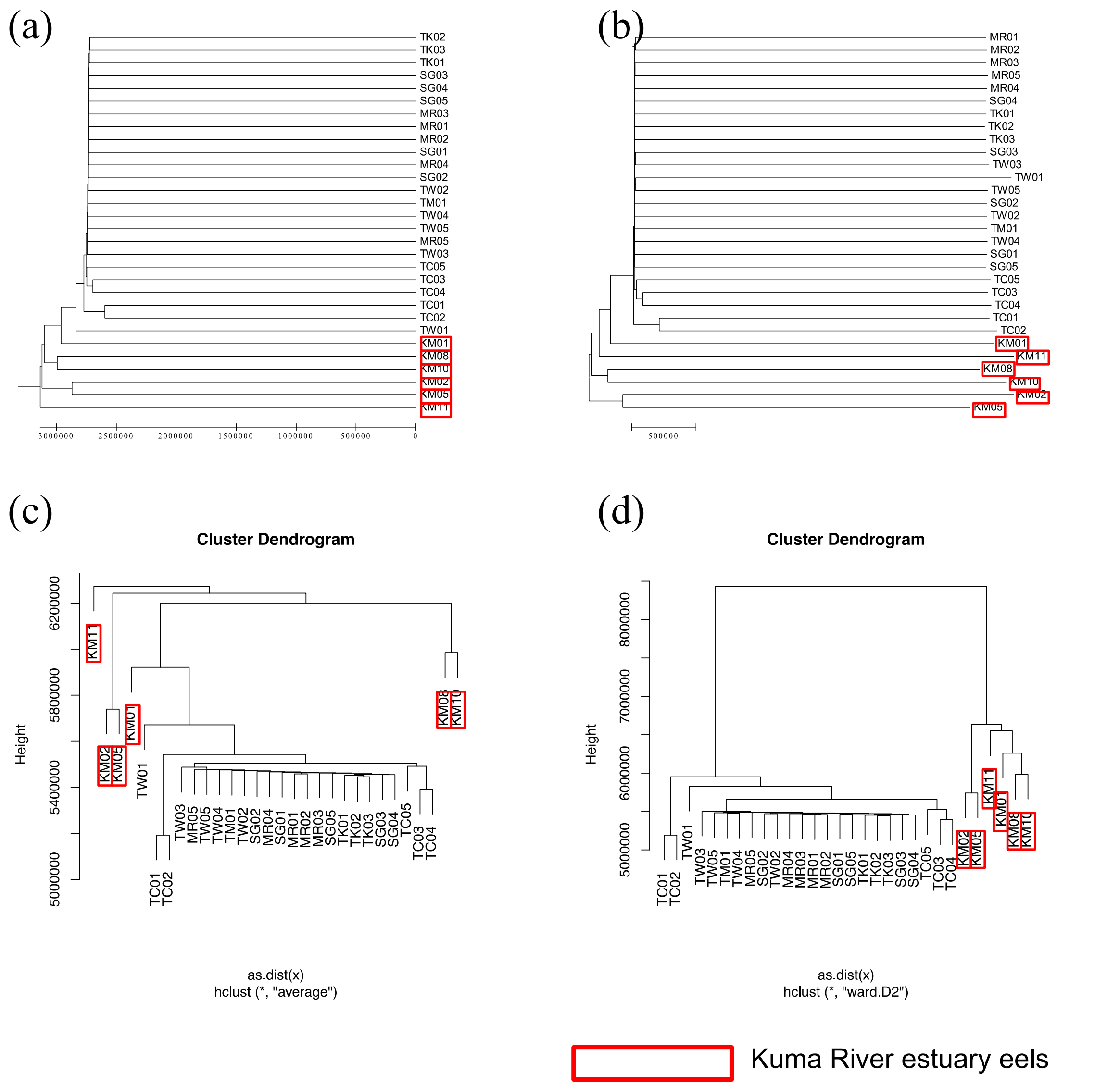

Figure S6. Molecular phylogenetic tree based on calculated pairwise genetic distance. Molecular phylogenetic tree constructed by the unweighted pair group method using MEGA6 software (**a**), the neighbor-joining method using MEGA6 software (**b**), the group average method using R (**c**), and the ward method using R (**d**) based on a pairwise genetic distance of 30 Japanese eel *Anguilla japonica* individuals.

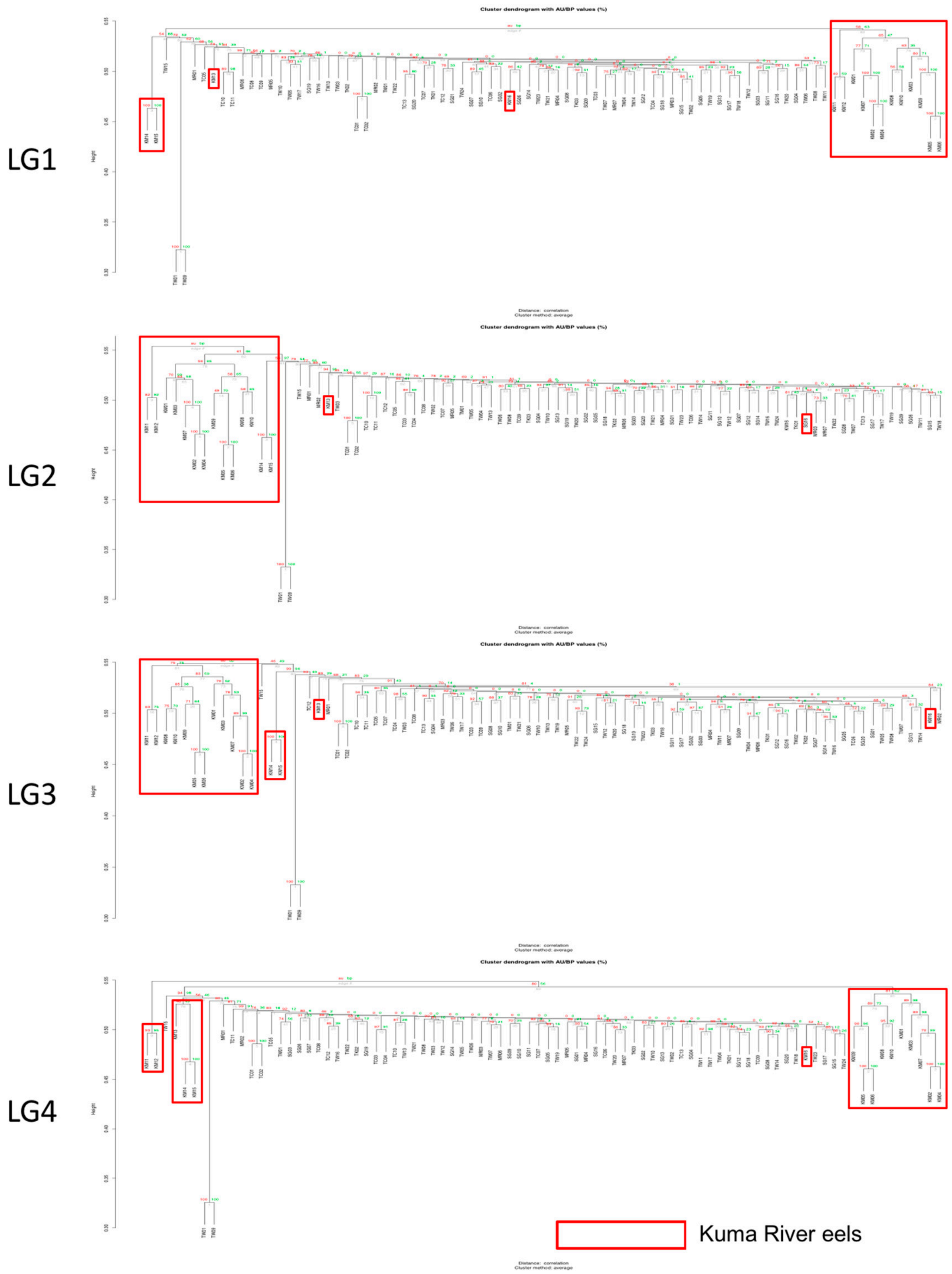

Figure S7. Genetic analysis of the 84 sampled Japanese eels *Anguilla japonica* based on SNP sites at different linkage groups of *A. japonica* ranged from 13,825 for LG17 (minimum) to 161,375 for LG9 (maximum). Results of the Cluster analyses and PCA using the SNP sites on each linkage group are shown.

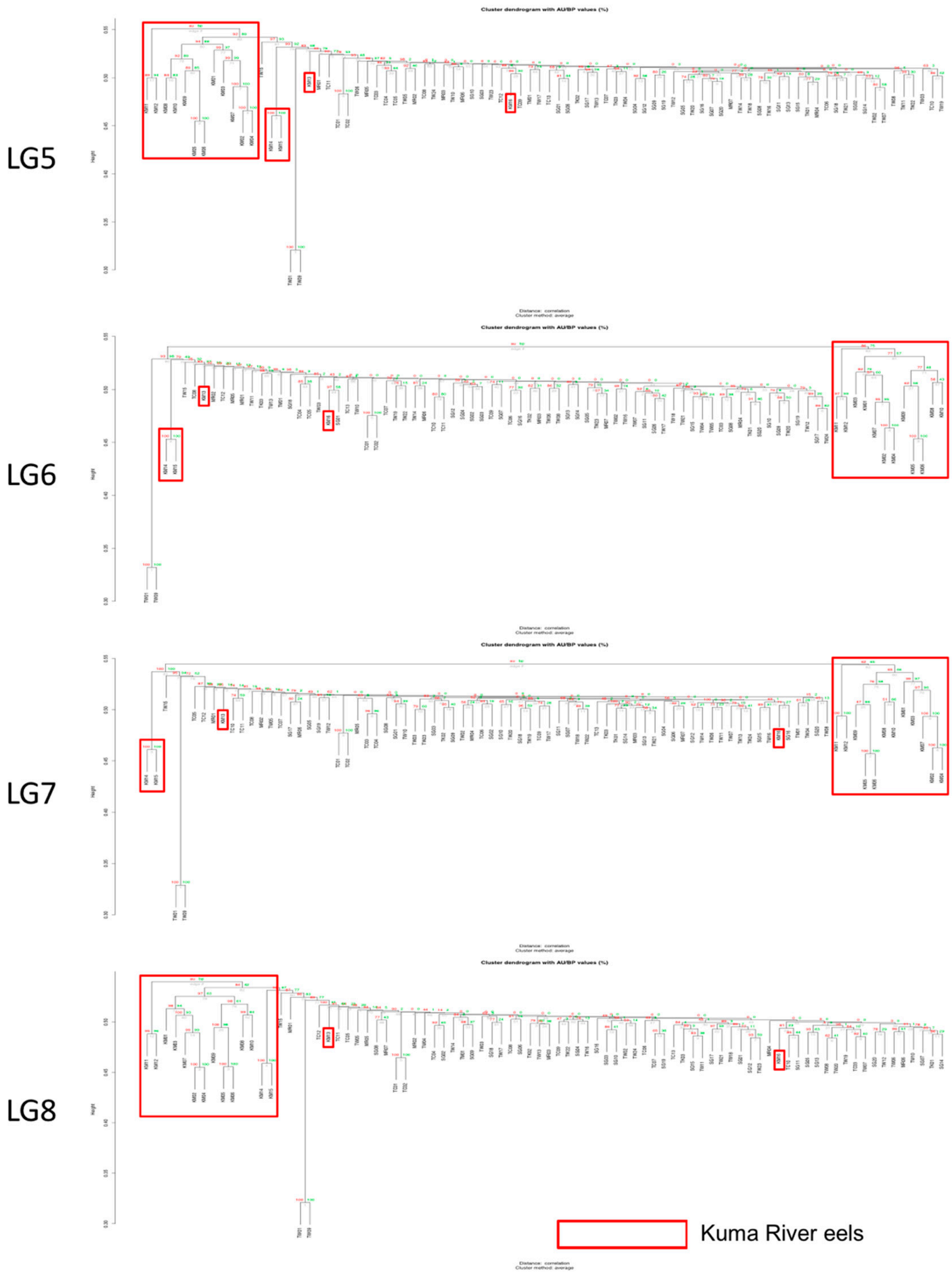

Figure S7. continued.

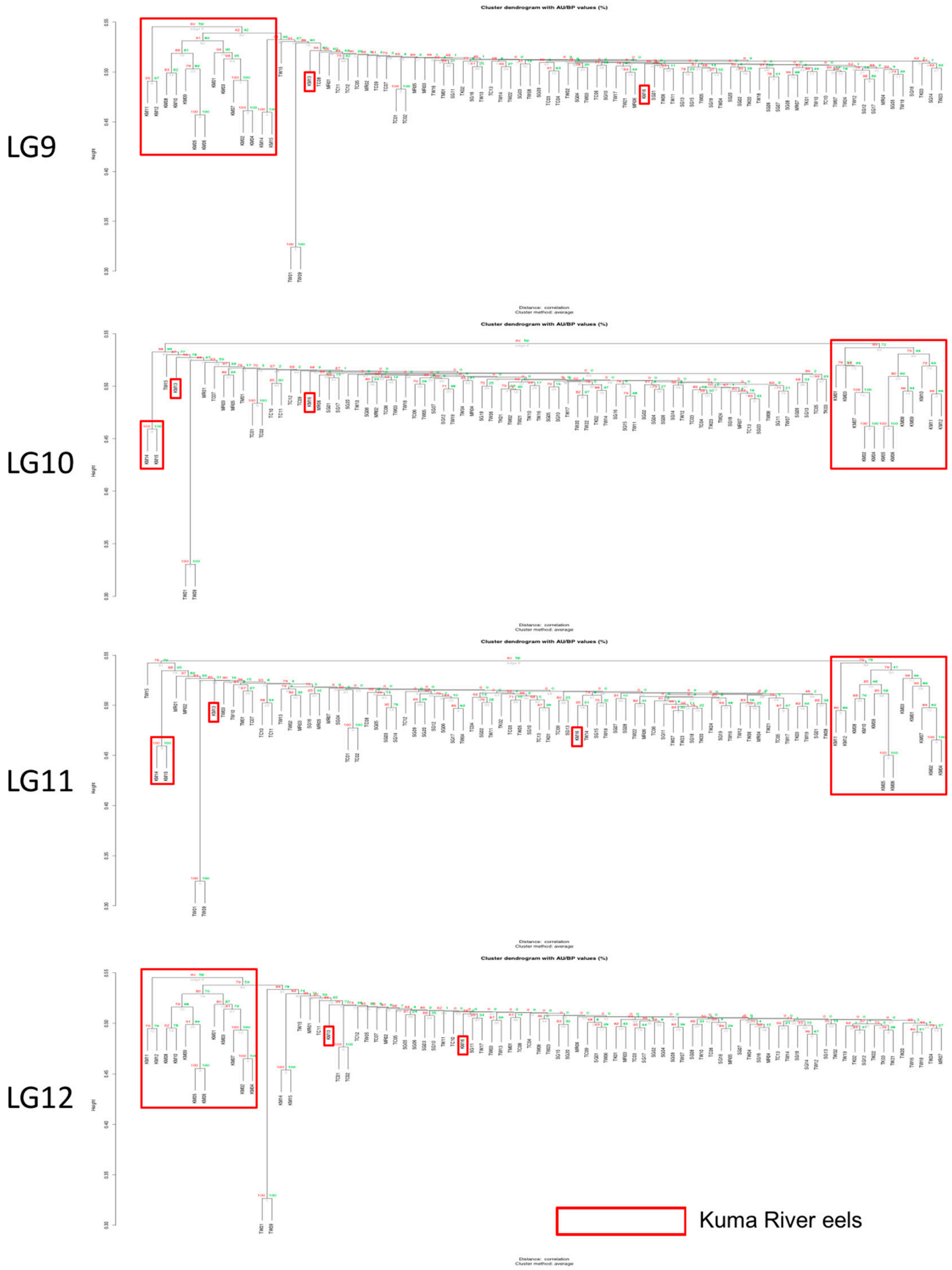

Figure S7. continued.

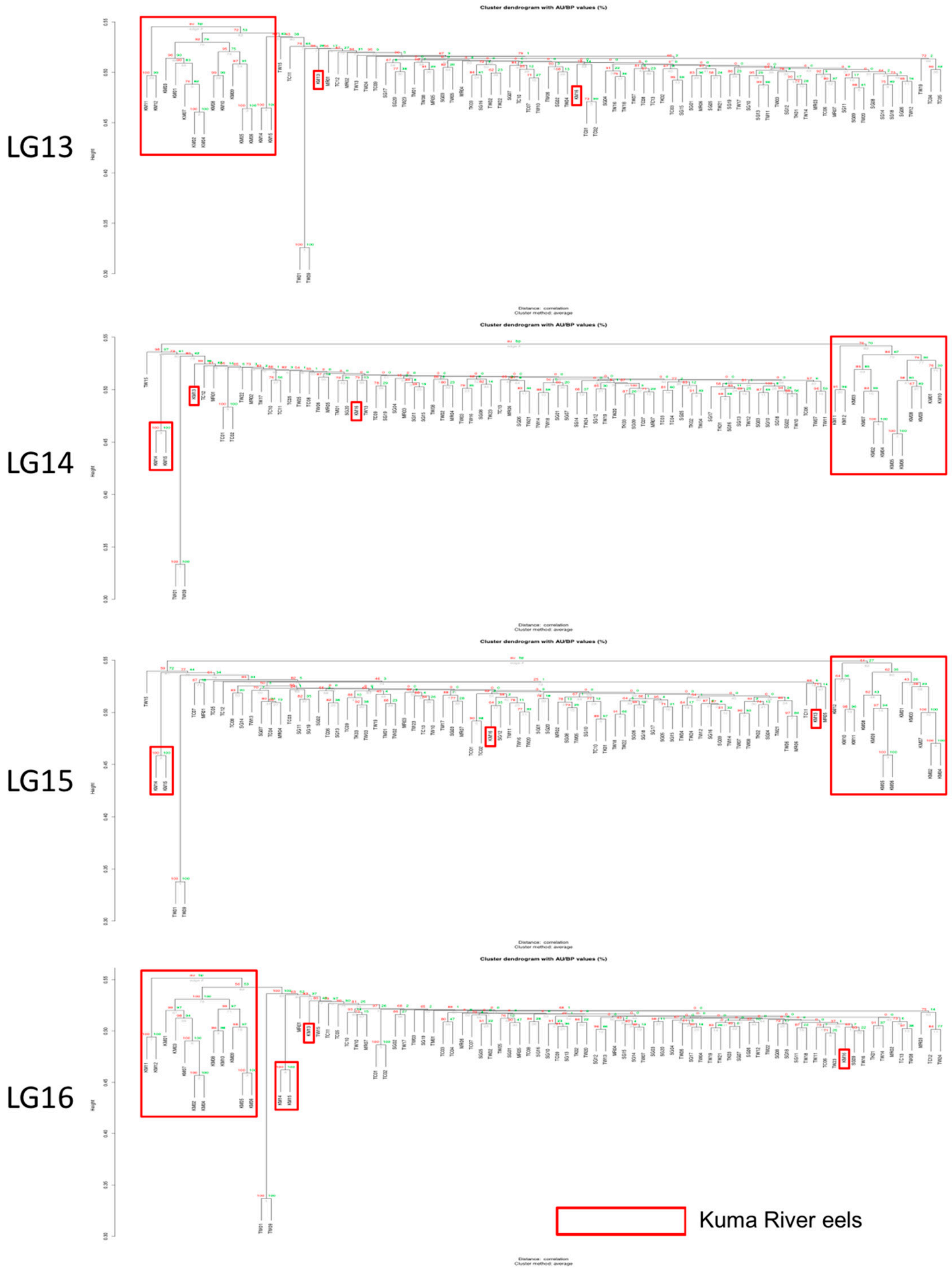

Figure S7. continued.

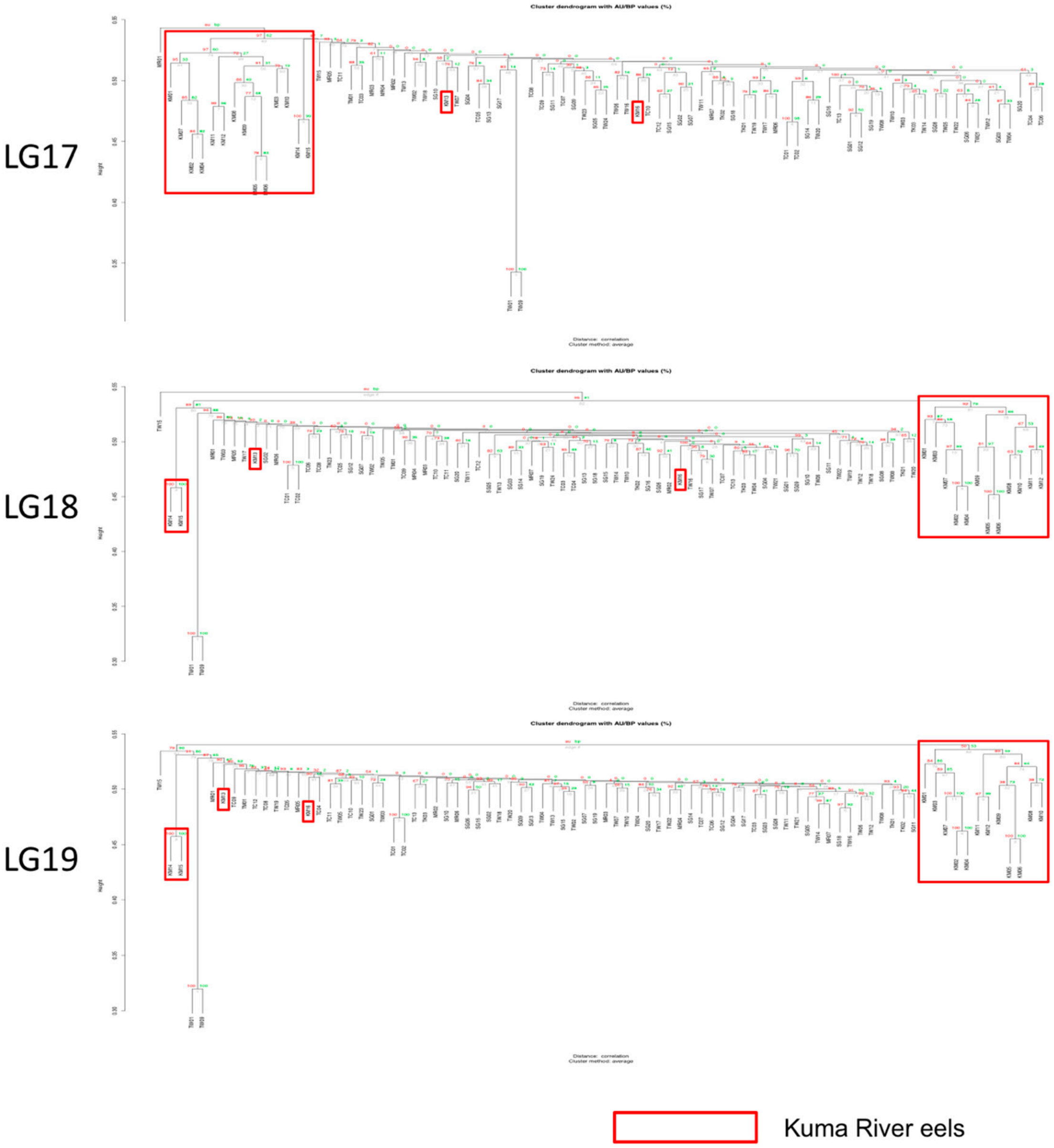

Figure S7. continued.

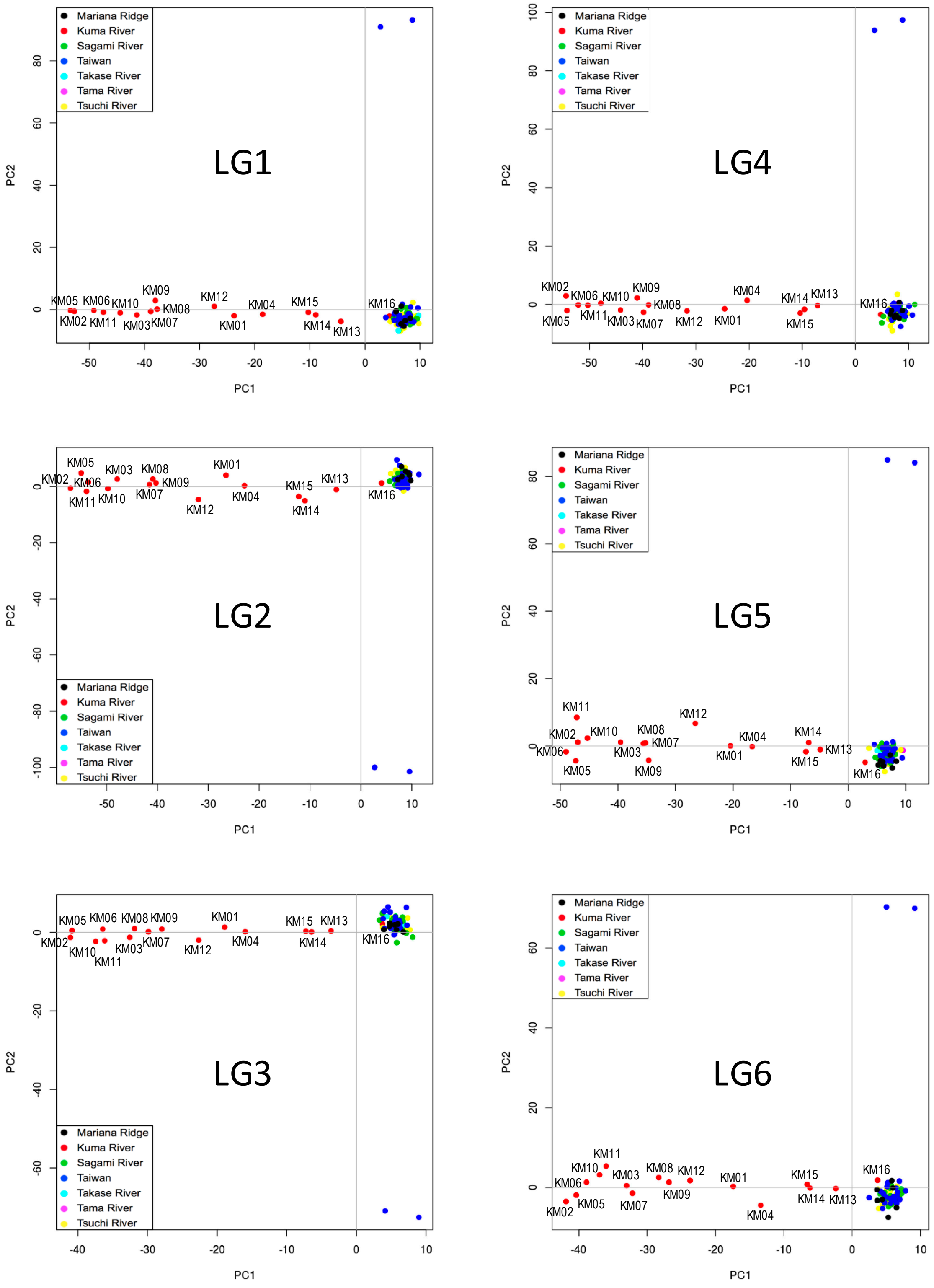

Figure S7. continued.

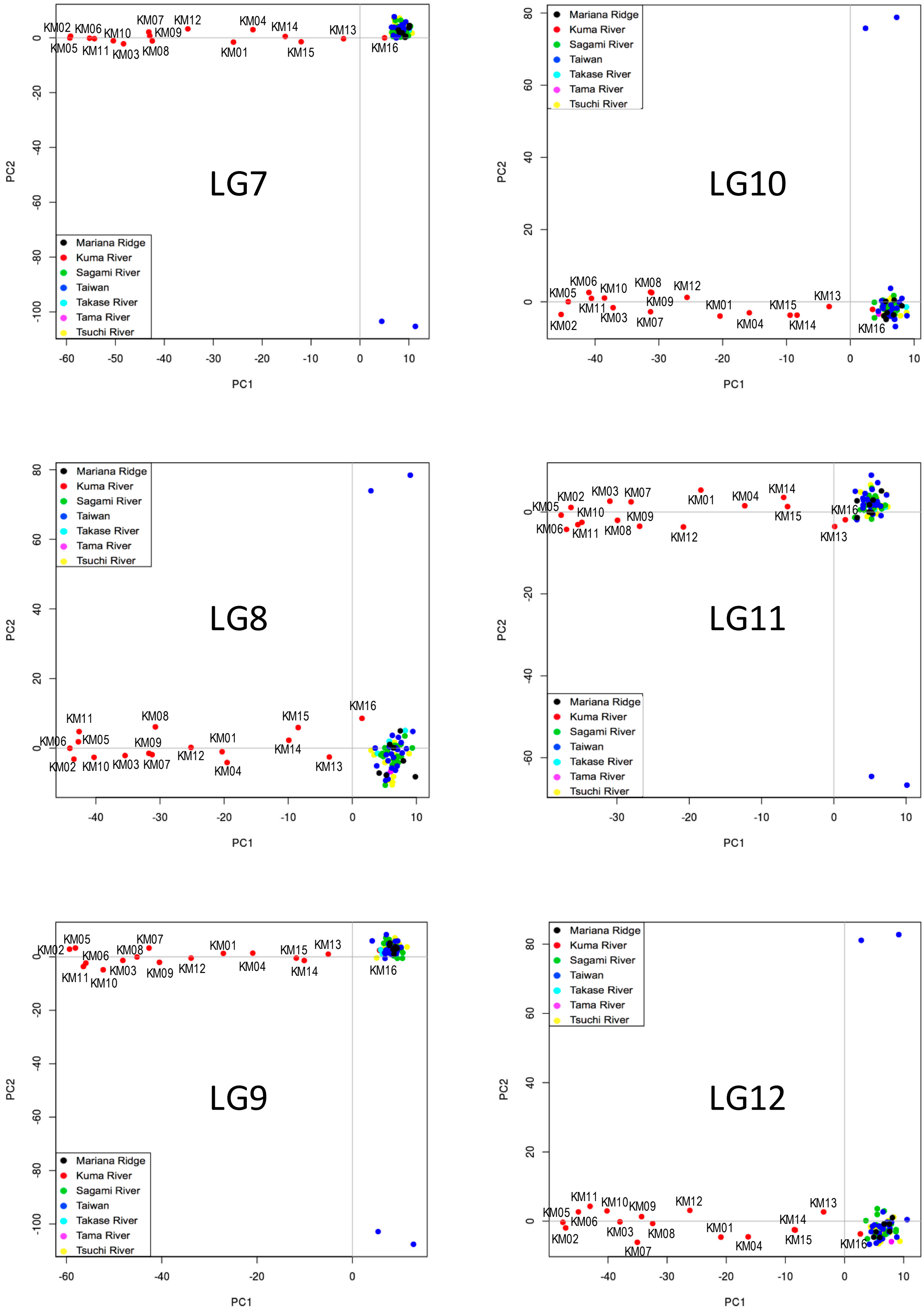

Figure S7. continued.

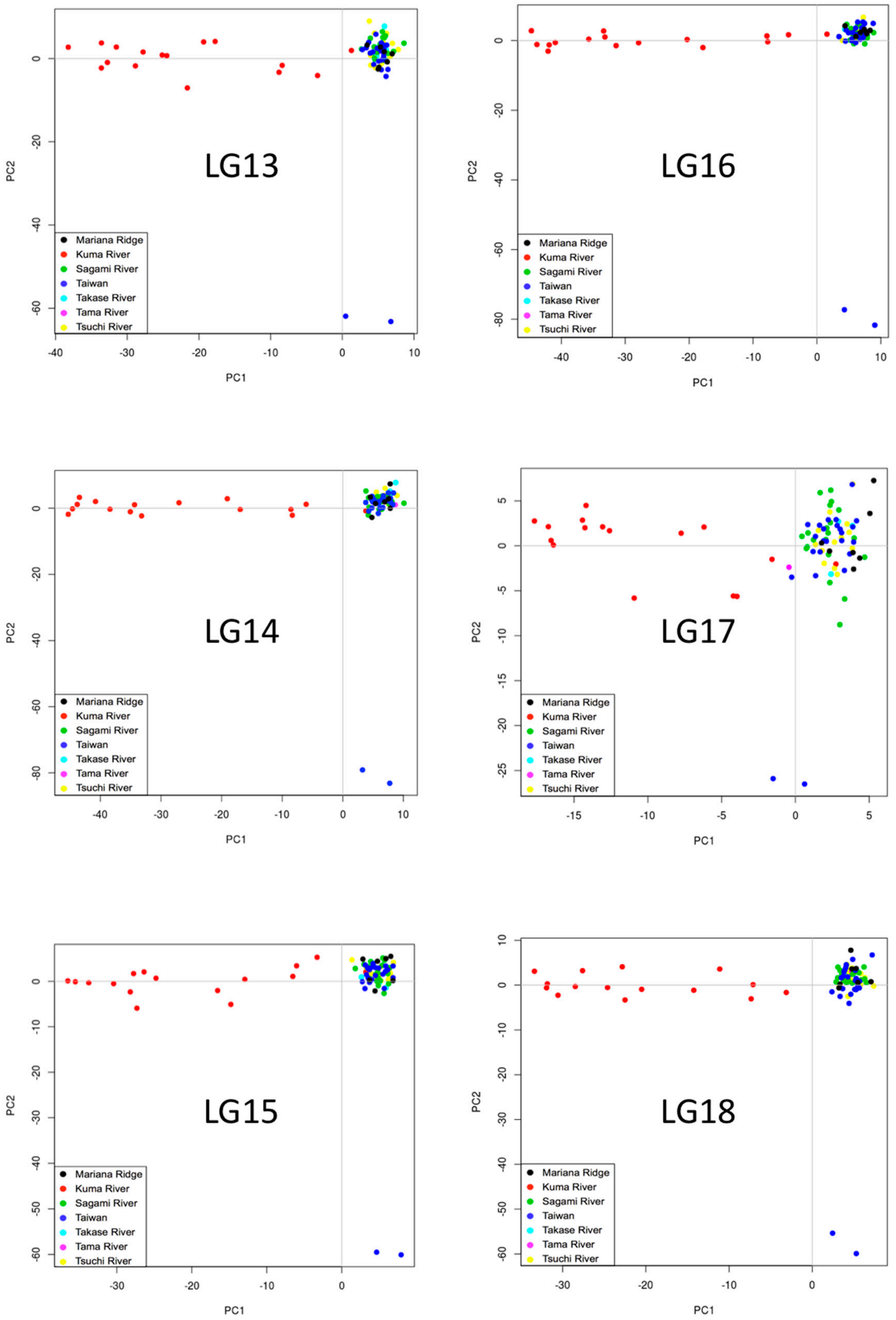

Figure S7. continued.

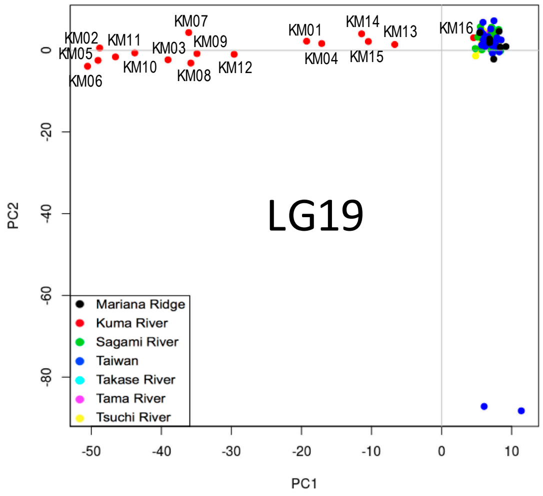

Figure S7. continued.

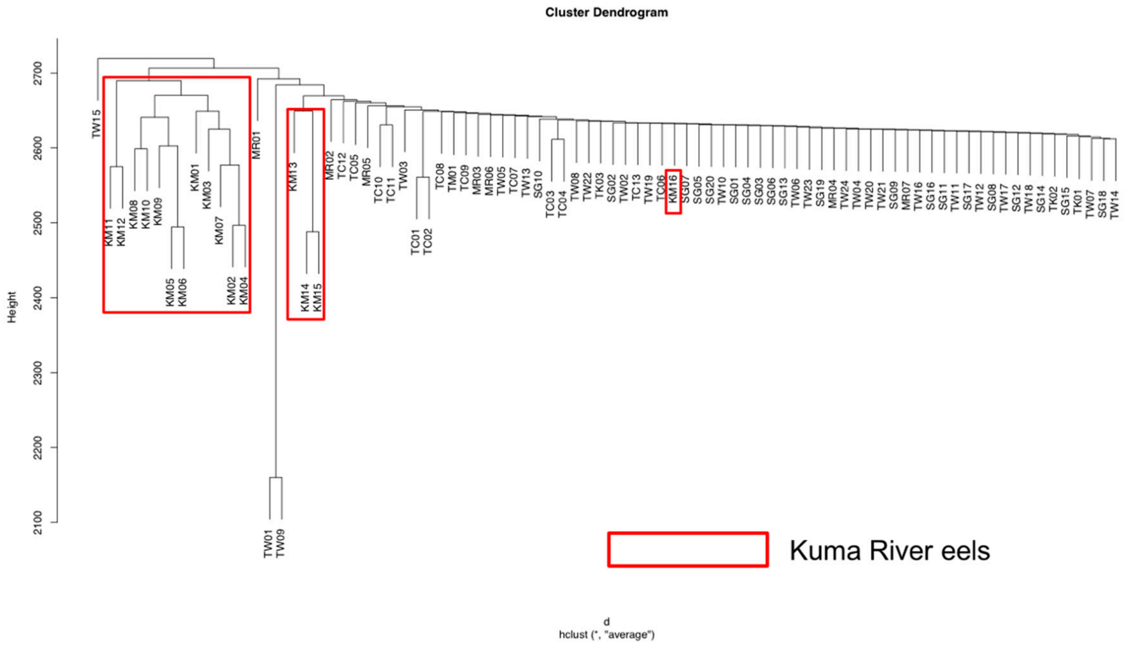

Figure S8. Cluster analysis of 84 Japanese eel *Anguilla japonica* individuals based on SNP sites extracted using another draft genome sequence of *A. japonica* assembled by ourselves as a reference (genome size: 1.16 Gb, number of scaffolds: 186743). A total of 34,587,126 SNP sites were detected using the same SNP filtering method (total sequencing depth 800-1100). The result was essentially the same as that obtained from the published reference genome in Figure 2.

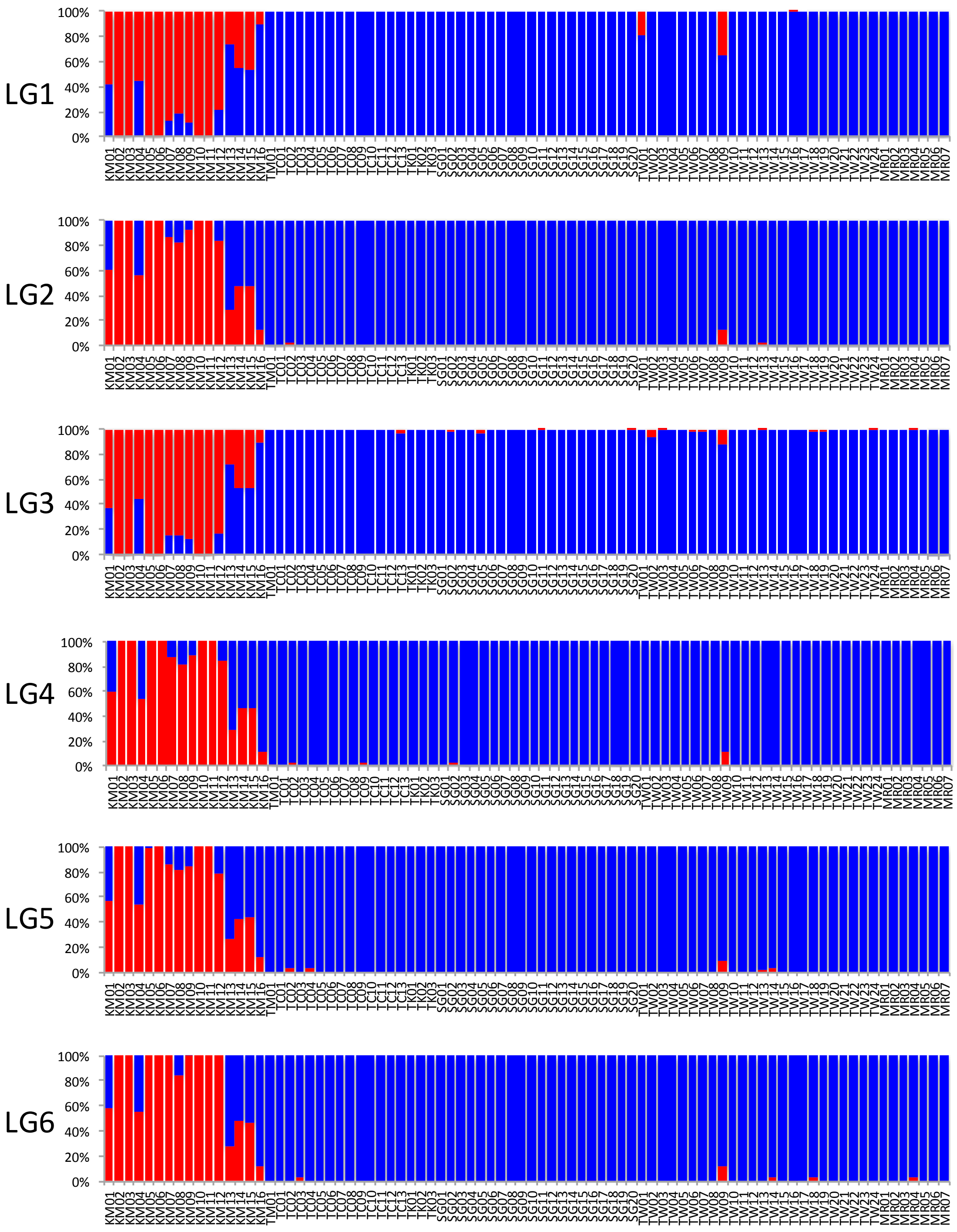

Figure S9. Population structure of the 84 Japanese eels by STRUCTURE software based on the SNP sites of each *Anguilla japonica* linkage group.

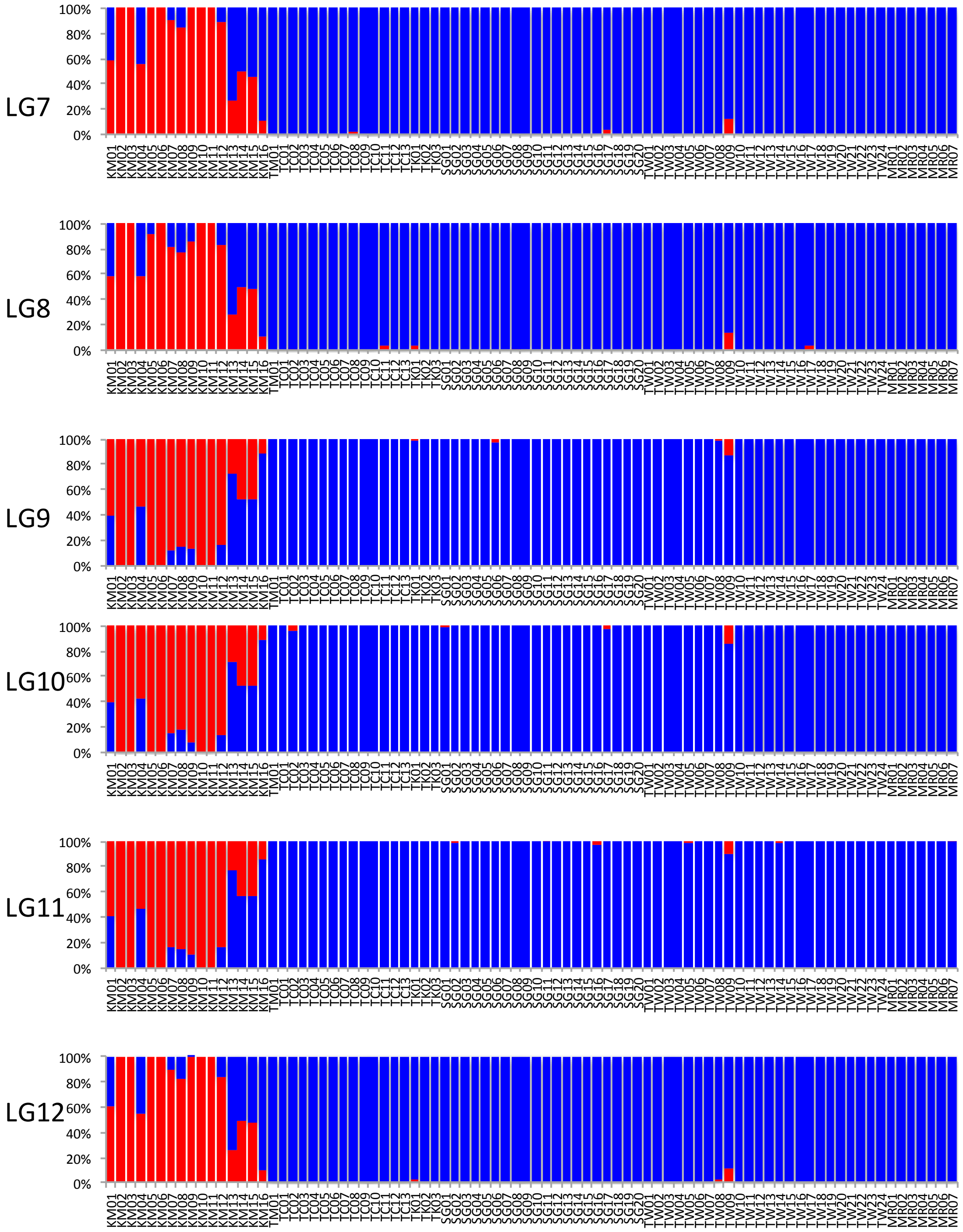

Figure S9. continued.

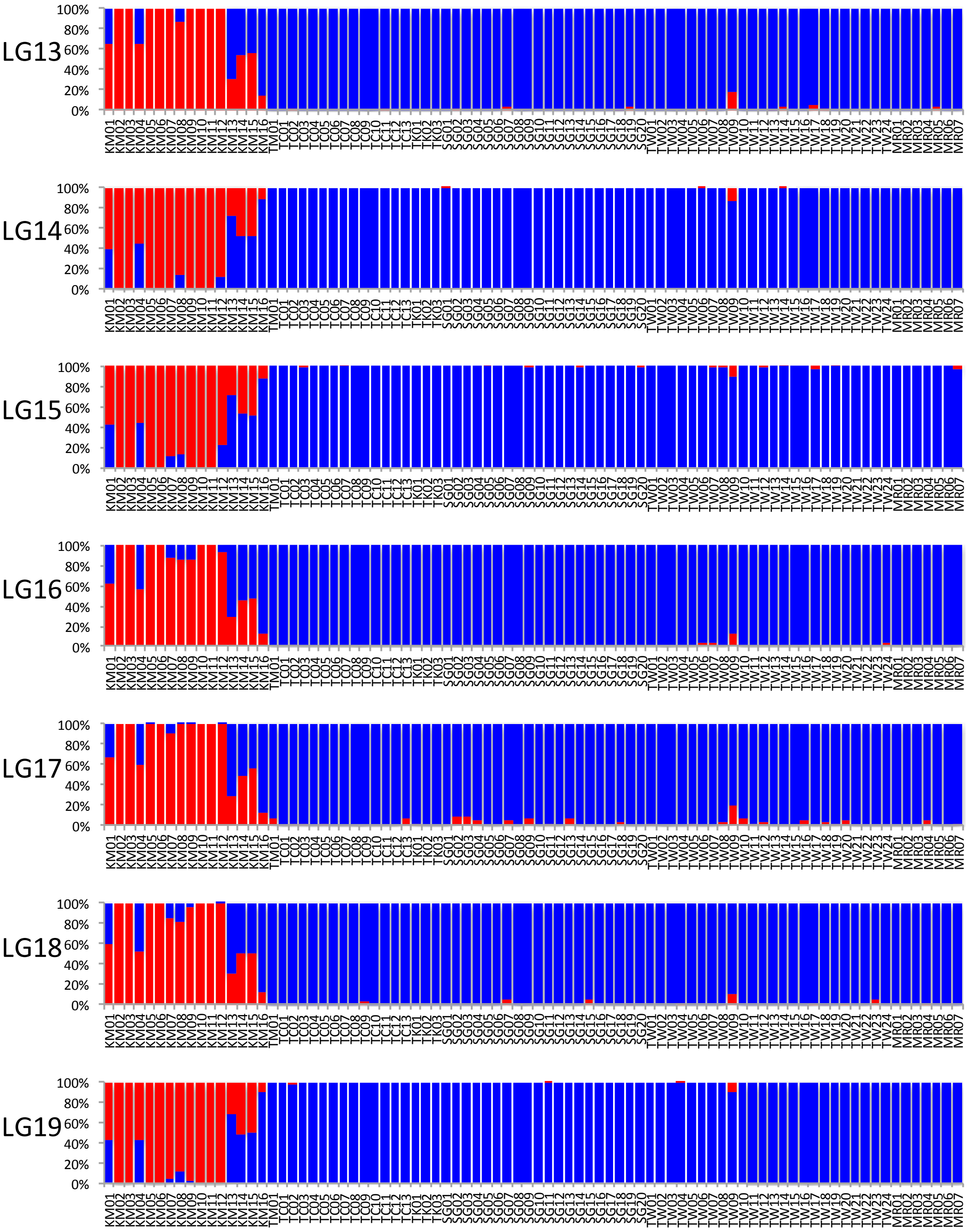

Figure S9. continued.

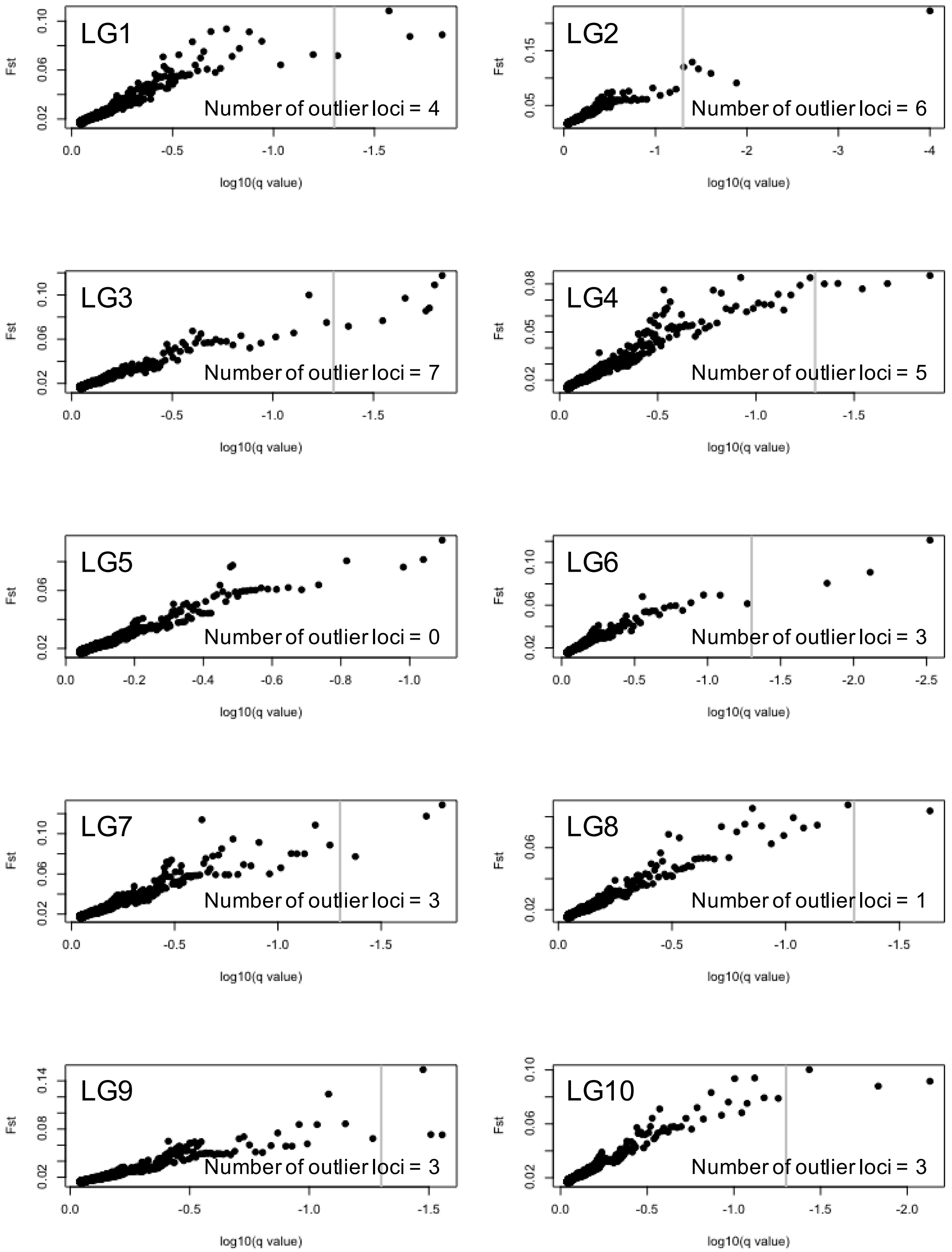

Figure S10. Detection of outlier SNPs in each linkage group using BayeScan. Log 10 (q value) is shown on the x-axis, and Fst-values on the y axis. The gray line indicates the Bayes factor threshold delineating 'very strong' evidence for selection (FDR > 0.05).

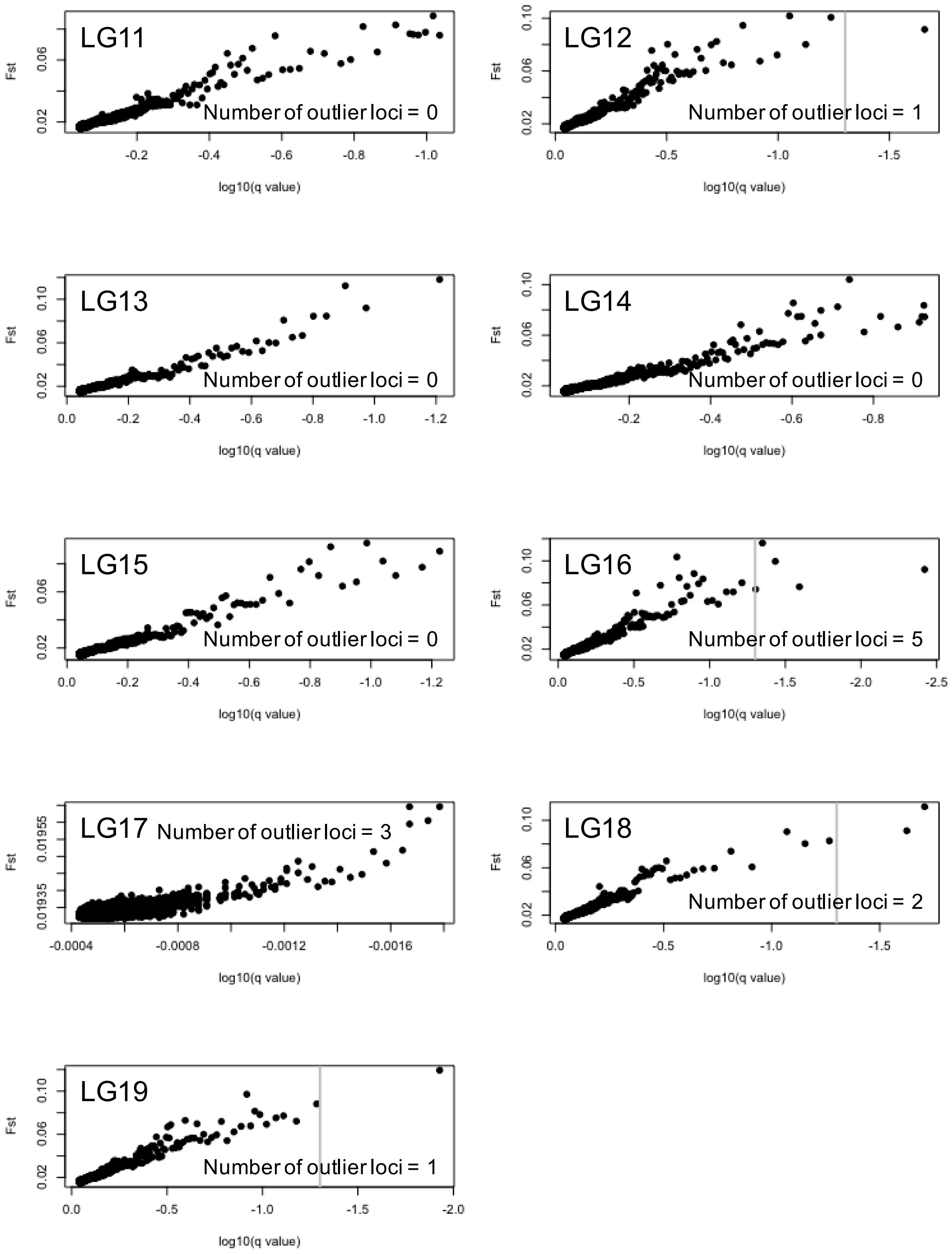

Figure S10. continued.

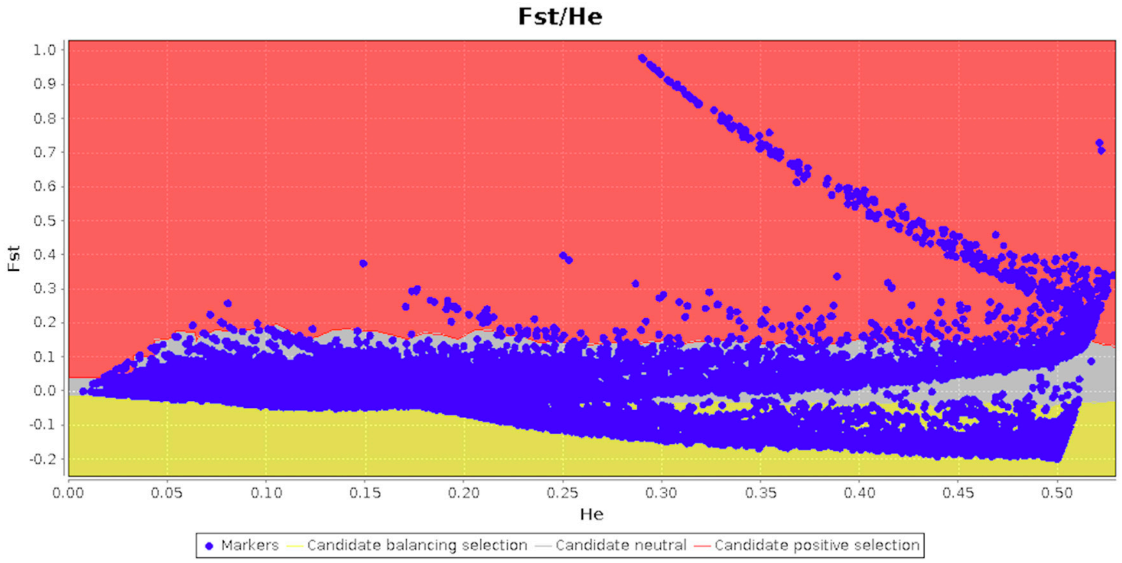

Figure S11. Detection of outlier SNPs in the 49,585 SNPs of LG18 using LOSITAN. The figure shows loci under positive selection above 99.5% percentile (red area), neutral loci (gray area) and loci under balancing selection (yellow area). 1,048 SNPs in the red zone are outlier SNPs and thus candidates for positive selection.

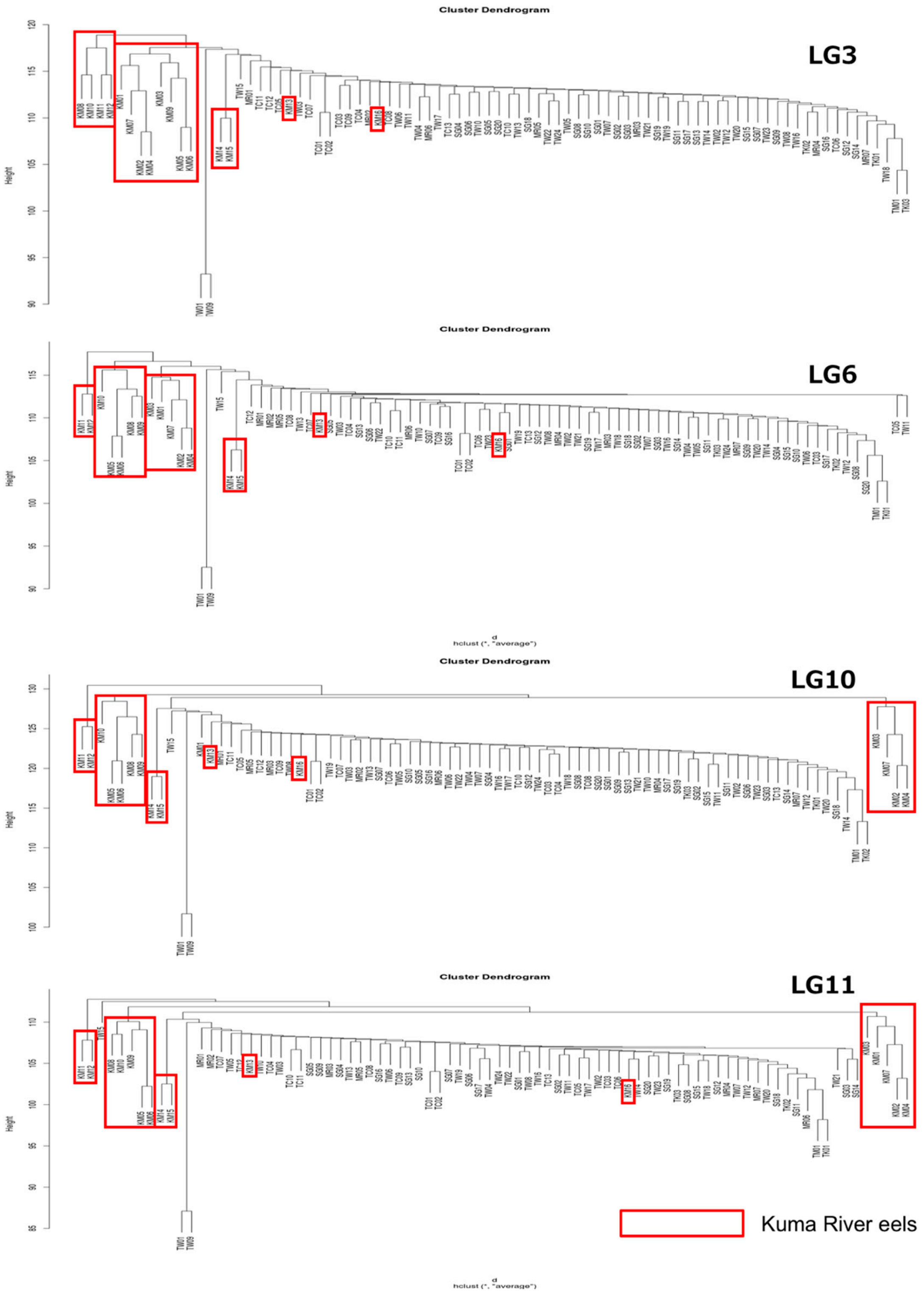

Figure S12. Cluster analysis and PCA of 84 sampled Japanese eels *Anguilla japonica,* based on SNP sites at different linkage groups of *A. japonica* without outlier SNP sites.

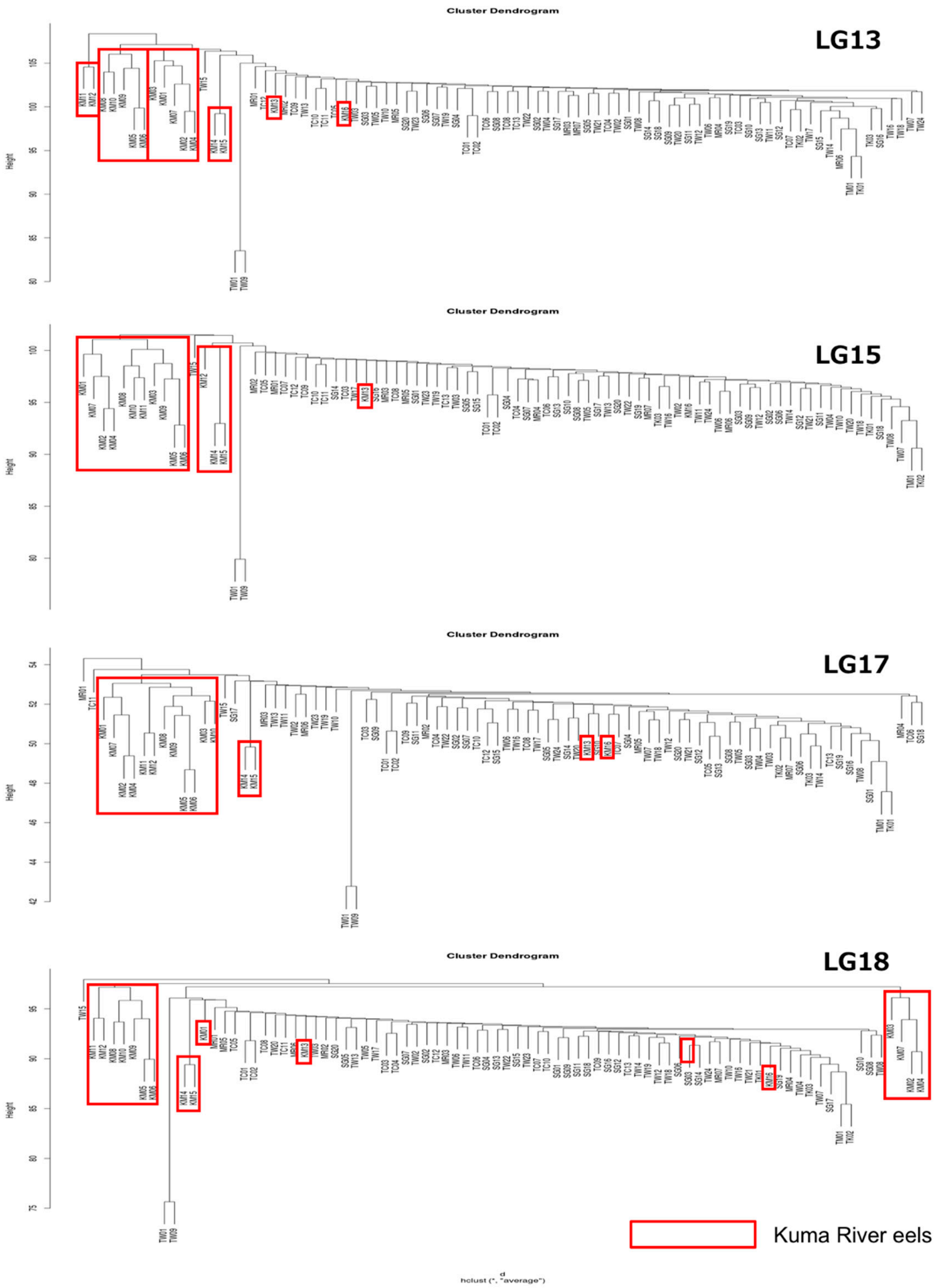

Figure S12. continued.

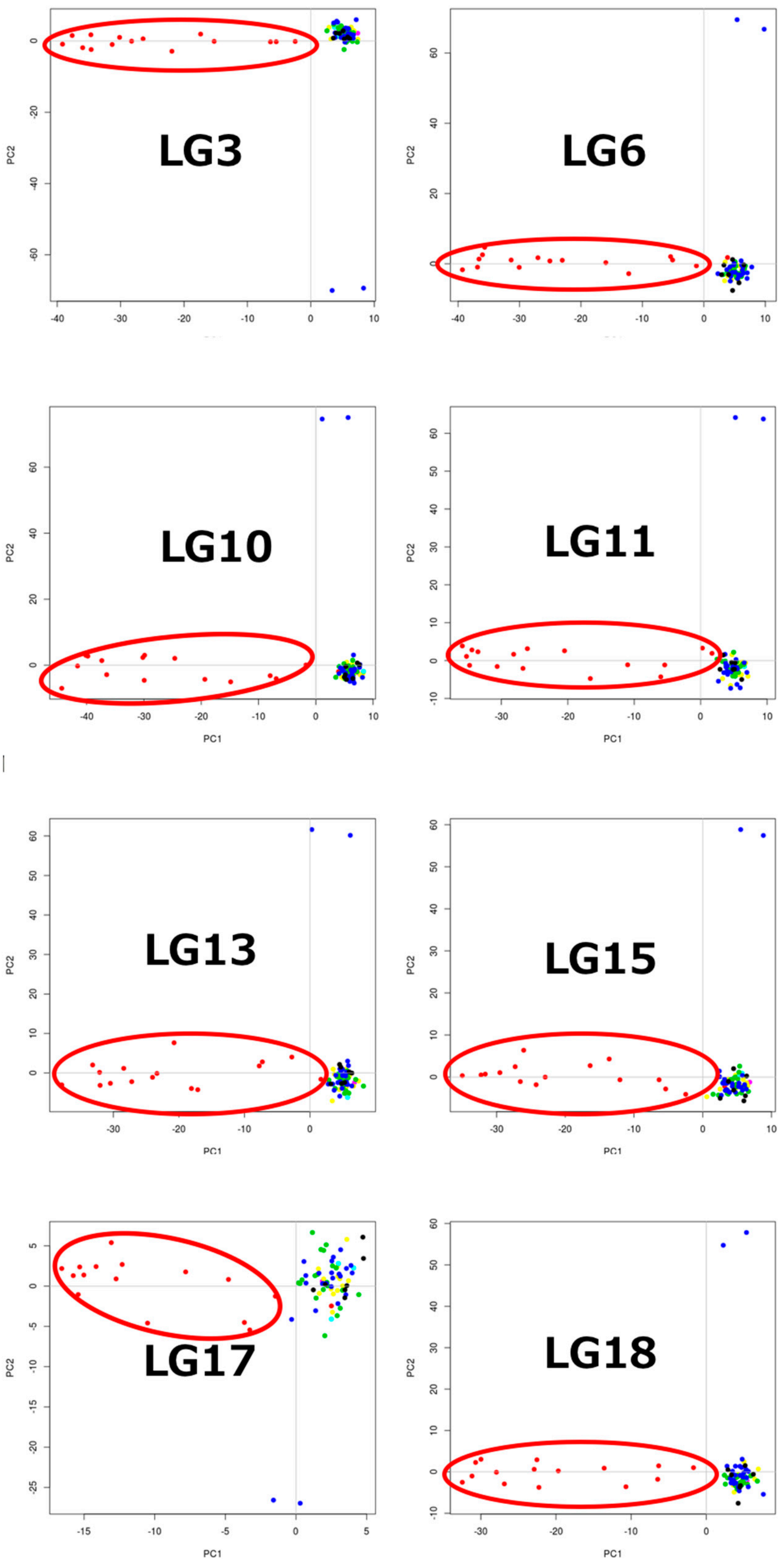

Figure S12. continued.

Table S1. Summary of the samples of *A. japonica* used in this study.

Sampling location	Sample ID	Total length (mm)	Body weight (g)	Age	Life stage	Sr/Ca ratio of otolith	Environment type	Date
Sagami-river, Kanagawa prefecture, Japan	SG01	59.0	-	154d	Glass eel	-	-	2013/4/9
	SG02	-	-	-	Glass eel	-	-	2013/4/9
	SG03	59.6	-	184d	Glass eel	-	-	2013/4/9
	SG04	58.7	-	167d	Glass eel	-	-	2013/4/9
	SG05	53.2	-	149d	Glass eel	-	-	2013/4/9
	SG06	54.6	-	124d	Glass eel	-	-	2013/4/9
	SG07	53.9	-	155d	Glass eel	-	-	2013/4/9
	SG08	54.4	-	146d	Glass eel	-	-	2013/4/9
	SG09	62.1	-	175d	Glass eel	-	-	2013/4/9
	SG10	59.1	-	139d	Glass eel	-	-	2013/4/9
	SG11	56.4	-	137d	Glass eel	-	-	2013/4/9
	SG12	57.7	-	143d	Glass eel	-	-	2013/4/9
	SG13	59.5	-	156d	Glass eel	-	-	2013/4/9
	SG14	55.4	-	132d	Glass eel	-	-	2013/4/9
	SG15	58.1	-	155d	Glass eel	-	-	2013/4/9
	SG16	60.8	-	157d	Glass eel	-	-	2013/4/9
	SG17	59.6	-	192d	Glass eel	-	-	2013/4/9
	SG18	55.2	-	166d	Glass eel	-	-	2013/4/9
	SG19	58.5	-	134d	Glass eel	-	-	2013/4/9
	SG20	53.5	-	147d	Glass eel	-	-	2013/4/9
Yilan City, North Taiwan	TW01	59.3	-	183d	Glass eel	-	-	2012/12/25
	TW02	-	-	-	Glass eel	-	-	2012/12/25
	TW03	57.8	-	104d	Glass eel	-	-	2012/12/25
	TW04	-	-	-	Glass eel	-	-	2012/12/25
	TW05	56.0	-	140d	Glass eel	-	-	2012/12/25
	TW06	56.1	-	156d	Glass eel	-	-	2012/12/25
	TW07	56.0	-	154d	Glass eel	-	-	2012/12/25
	TW08	60.1	-	117d	Glass eel	-	-	2012/12/25
	TW09	55.0	-	185d	Glass eel	-	-	2012/12/25
	TW10	53.1	-	142d	Glass eel	-	-	2012/12/25
	TW11	60.4	-	126d	Glass eel	-	-	2012/12/25
	TW12	57.5	-	109d	Glass eel	-	-	2012/12/25
	TW13	56.0	-	122d	Glass eel	-	-	2012/12/25
	TW14	60.0	-	155d	Glass eel	-	-	2012/12/25
	TW15	59.1	-	181d	Glass eel	-	-	2012/12/25
	TW16	61.4	-	131d	Glass eel	-	-	2012/12/25
	TW17	54.9	-	135d	Glass eel	-	-	2012/12/25
	TW18	59.0	-	124d	Glass eel	-	-	2012/12/25
	TW19	58.8	-	122d	Glass eel	-	-	2012/12/25
	TW20	59.9	-	147d	Glass eel	-	-	2012/12/25
	TW21	55.6	-	140d	Glass eel	-	-	2012/12/25
	TW22	52.9	-	158d	Glass eel	-	-	2012/12/25
	TW23	-	-	-	Glass eel	-	-	2012/12/25
	TW24	-	-	-	Glass eel	-	-	2012/12/25
Kuma River estuary, Kumamoto prefecture, Japan	KM01	628	385	5+	Yellow to silver eel	4.6	Brackish	2011/10/15
	KM02	661	388	7+	Yellow to silver eel	6.2	Sea	2011/10/15
	KM03	600	320	-	Yellow to silver eel	-	-	2011/10/15
	KM04	612	301	-	Yellow to silver eel	4.9	Brackish	2011/10/15
	KM05	584	280	-	Yellow to silver eel	-	-	2011/10/15
	KM06	668	390	6+	Yellow to silver eel	5.9	Brackish	2011/10/15
	KM07	641	335	4+	Yellow to silver eel	5.5	Brackish	2011/10/15
	KM08	563	231	4+	Yellow to silver eel	5.6	Brackish	2011/10/15
	KM09	591	262	5+	Yellow to silver eel	6.3	Sea	2011/10/15
	KM10	646	408	8+	Yellow to silver eel	5.6	Brackish	2011/10/15
	KM11	511	134	7+	Yellow to silver eel	6.3	Fresh	2011/8/17
	KM12	481	102	5+	Yellow to silver eel	5.7	Brackish	2011/8/17
	KM13	470	101	5+	Yellow to silver eel	5.9	Brackish	2011/8/17
	KM14	434	75	6+	Yellow to silver eel	4.6	Brackish	2011/8/17
	KM15	395	50	4+	Yellow to silver eel	6.3	Sea	2011/8/17
	KM16	425	70	4+	Yellow to silver eel	6.4	Sea	2011/8/17
Tama River estuary, Kanagawa Prefecture, Japan	TM01	735	628	-	Yellow to silver eel	5.7	Brackish	2011/8/8
Tsuchi River, Kagoshima Prefecture, Japan	TC01	269	18	-	Yellow to silver eel	2.0	Fresh	2013/8/2
	TC02	505	168	-	Yellow to silver eel	2.1	Fresh	2013/8/2
	TC03	276	23	-	Yellow to silver eel	2.1	Fresh	2013/8/2
	TC04	340	53	-	Yellow to silver eel	1.8	Fresh	2013/8/2
	TC05	321	39	-	Yellow to silver eel	1.6	Fresh	2013/8/2
	TC06	362	59	-	Yellow to silver eel	1.9	Fresh	2013/8/2
	TC07	191	8	-	Yellow to silver eel	2.2	Fresh	2013/8/2
	TC08	276	27	-	Yellow to silver eel	2.8	Brackish	2013/8/2
	TC09	485	147	-	Yellow to silver eel	3.1	Brackish	2013/8/2
	TC10	636	373	-	Yellow to silver eel	2.6	Brackish	2013/11/19
	TC11	308	29	-	Yellow to silver eel	1.9	Fresh	2013/11/19
	TC12	422	91	-	Yellow to silver eel	2.4	Fresh	2013/11/19
	TC13	465	134	-	Yellow to silver eel	3.0	Brackish	2013/11/19
Takase River, Wakayama Prefecture, Japan	TK01	225	225	-	Yellow eel	1.9	Fresh	2014/7/2
	TK02	526	526	-	Yellow eel	2.8	Brackish	2014/9/30
	TK03	290	290	-	Yellow eel	2.7	Brackish	2014/9/30
West Mariana Ridge of the Pacific Ocean	MR01	560	118	7+	Silver eel	-	-	2013/6/11
	MR02	683	202	6+	Silver eel	-	-	2013/6/11
	MR03	616	128	10+	Silver eel	-	-	2013/6/11
	MR04	532	185	9	Silver eel	-	-	2013/6/12
	MR05	729	166	6	Silver eel	-	-	2013/7/9
	MR06	471	83	6+	Silver eel	-	-	2009/6/12
	MR07	739	330	10+	Silver eel	-	-	2009/6/24

Table S2. The score matrix to calculate the pairwise distance. K, G or T. M, A or C. S, C or G. R, A or G. W, A or T. Y, C or T. N, A or C or G or T.

	A	C	G	T	K	M	S	R	W	Y	N
A	0	2	2	2	2	1	2	1	1	2	0
C	2	0	2	2	2	1	1	2	2	1	0
G	2	2	0	2	1	2	1	1	2	2	0
T	2	2	2	0	1	2	2	2	1	1	0
K	2	2	1	1	0	2	1	1	1	1	0
M	1	1	2	2	2	0	1	1	1	1	0
S	2	1	1	2	1	1	0	1	2	1	0
R	1	2	1	2	1	1	1	0	1	2	0
W	1	2	2	1	1	1	2	1	0	1	0
Y	2	1	2	1	1	1	1	2	1	0	0
N	0	0	0	0	0	0	0	0	0	0	0

Table S3. Summary of sequence data obtained for each sample.

* The depth of coverage was estimated from total residues of sequence / genome size of Japanese eel (1.15 Gb).

Sampling location	Sample ID	Raw reads	Raw residues (bp)	Raw depth of coverage*	High quality reads	High quality residues (bp)	High quality depth of coverage*	Mapped reads	Mapped ratio	Mapped residues (bp)	Actual depth of coverage*
Sagami River	SG01	160,007,738	15,967,748,832	13.9	155,172,037	15,470,861,341	13.5	137,330,406	88.5	13,692,026,671	11.9
	SG02	146,649,316	14,612,304,208	12.7	141,676,353	14,105,180,824	12.3	125,376,269	88.5	12,482,357,908	10.9
	SG03	152,728,632	15,152,582,680	13.2	147,643,403	14,636,151,388	12.7	130,963,523	88.7	12,982,645,414	11.3
	SG04	156,085,548	15,372,025,872	13.4	145,420,748	14,319,677,283	12.5	128,753,227	88.5	12,678,415,461	11.0
	SG05	156,152,544	15,357,835,224	13.4	146,608,257	14,415,182,877	12.5	129,997,835	88.7	12,781,971,517	11.1
	SG06	152,982,426	15,061,394,216	13.1	143,462,249	14,118,501,391	12.3	127,186,168	88.7	12,516,728,982	10.9
	SG07	146,061,748	14,398,449,240	12.5	136,094,494	13,415,340,376	11.7	120,717,426	88.7	11,899,565,600	10.3
	SG08	211,785,968	21,178,596,800	18.4	196,928,342	19,660,027,958	17.1	173,672,628	88.2	17,338,330,721	15.1
	SG09	188,851,900	18,885,190,000	16.4	183,339,354	18,313,452,521	15.9	160,759,084	87.7	16,057,948,214	14.0
	SG10	139,112,740	13,854,568,744	12.0	133,200,839	13,253,599,264	11.5	117,908,468	88.5	11,731,995,057	10.2
	SG11	187,193,148	18,719,314,800	16.3	182,394,414	18,220,813,533	15.8	160,431,495	88.0	16,026,764,697	13.9
	SG12	269,351,798	26,841,294,944	23.3	244,103,238	24,262,546,850	21.1	215,544,903	88.3	21,424,002,197	18.6
	SG13	163,493,424	15,989,278,160	13.9	153,082,339	14,940,919,442	13.0	135,693,865	88.6	13,243,794,934	11.5
	SG14	231,395,626	22,970,603,776	20.0	214,473,973	21,238,705,443	18.5	188,630,672	88.0	18,679,521,921	16.2
	SG15	252,100,734	24,975,986,968	21.7	229,600,841	22,682,835,943	19.7	202,567,316	88.2	20,012,127,030	17.4
	SG16	199,201,706	19,564,788,240	17.0	187,755,988	18,411,057,663	16.0	166,155,098	88.5	16,292,908,273	14.2
	SG17	226,913,822	22,428,656,096	19.5	201,530,727	19,847,808,774	17.3	177,426,210	88.0	17,473,868,824	15.2
	SG18	410,309,516	39,389,713,536	34.3	393,382,895	37,694,861,475	32.8	344,975,938	87.7	33,056,394,572	28.7
	SG19	207,023,090	20,396,684,600	17.7	182,499,425	17,904,832,809	15.6	160,655,954	88.0	15,761,792,105	13.7
	SG20	167,144,838	16,045,904,448	14.0	157,190,639	15,063,685,652	13.1	138,762,687	88.3	13,297,722,501	11.6
Taiwan	TW01	142,676,022	14,172,379,256	12.3	137,804,526	13,677,329,344	11.9	85,613,815	62.1	8,497,314,117	7.4
	TW02	147,687,236	14,640,860,264	12.7	143,831,630	14,248,494,720	12.4	127,110,958	88.4	12,592,082,937	10.9
	TW03	116,041,826	11,604,182,600	10.1	113,869,682	11,376,975,919	9.9	100,449,752	88.2	10,036,160,543	8.7
	TW04	172,105,420	17,210,542,000	15.0	166,756,633	16,655,805,039	14.5	147,990,790	88.7	14,781,455,475	12.9
	TW05	158,991,570	15,864,480,088	13.8	154,630,367	15,411,891,872	13.4	130,974,714	84.7	13,054,150,807	11.4
	TW06	214,450,772	21,445,077,200	18.6	200,814,958	20,043,997,904	17.4	142,507,043	71.0	14,224,094,159	12.4
	TW07	251,595,362	25,159,536,200	21.9	239,545,775	23,922,961,490	20.8	212,048,979	88.5	21,176,911,004	18.4
	TW08	146,160,282	14,485,151,232	12.6	140,651,629	13,925,887,740	12.1	124,575,884	88.6	12,334,231,662	10.7
	TW09	227,364,140	22,736,414,000	19.8	207,209,822	20,686,478,530	18.0	162,909,823	78.6	16,263,855,271	14.1
	TW10	175,045,178	17,504,517,800	15.2	170,814,405	17,063,974,170	14.8	146,136,299	85.6	14,598,686,987	12.7
	TW11	183,201,526	18,320,152,600	15.9	178,971,391	17,879,170,416	15.5	157,824,820	88.2	15,766,636,427	13.7
	TW12	241,000,556	23,813,532,840	20.7	216,570,603	21,328,167,989	18.5	191,088,551	88.2	18,818,660,797	16.4
	TW13	150,101,364	14,409,730,944	12.5	140,241,268	13,430,764,214	11.7	120,657,600	86.0	11,555,256,162	10.0
	TW14	301,106,610	29,806,701,688	25.9	278,993,434	27,550,825,293	24.0	186,322,848	66.8	18,399,530,626	16.0
	TW15	95,488,190	9,166,866,240	8.0	92,987,838	8,918,113,967	7.8	78,479,290	84.4	7,526,653,671	6.5
	TW16	188,967,656	18,567,288,448	16.1	182,791,176	17,945,647,012	15.6	161,493,152	88.3	15,854,699,138	13.8
	TW17	194,682,324	19,312,818,384	16.8	181,350,875	17,951,341,114	15.6	160,435,262	88.5	15,880,971,707	13.8
	TW18	241,063,108	23,868,510,736	20.8	220,737,468	21,791,716,807	18.9	190,071,481	86.1	18,764,299,168	16.3
	TW19	186,474,174	18,656,595,280	16.2	171,641,569	16,939,644,195	14.7	138,113,663	80.5	13,630,709,177	11.9
	TW20	178,950,022	17,580,748,976	15.3	171,598,755	16,834,374,012	14.6	152,172,027	88.7	14,928,551,298	13.0
	TW21	237,709,984	23,487,841,352	20.4	210,850,215	20,755,886,176	18.0	169,041,859	80.2	16,640,313,051	14.5
	TW22	164,151,692	15,758,562,432	13.7	151,717,823	14,542,858,701	12.6	133,444,112	88.0	12,791,238,544	11.1
	TW23	192,338,700	18,888,833,648	16.4	180,176,183	17,659,658,209	15.4	158,617,664	88.0	15,546,637,105	13.5
	TW24	173,835,938	17,061,435,752	14.8	166,874,885	16,356,040,155	14.2	147,871,789	88.6	14,493,474,669	12.6

Table S3. continued.

Sampling location	Sample ID	Raw reads	Raw residues (bp)	Raw depth of coverage*	High quality reads	High quality residues (bp)	High quality depth of coverage*	Mapped reads	Mapped ratio	Mapped residues (bp)	Actual depth of coverage*
Kuma River estuary	KM01	158,288,906	15,744,270,726	13.7	138,387,602	13,681,566,795	11.9	122,178,822	88.3	12,079,100,223	10.5
	KM02	168,910,616	16,720,555,966	14.5	145,734,939	14,347,593,388	12.5	128,230,942	88.0	12,624,326,247	11.0
	KM03	195,365,358	19,542,109,858	17.0	165,987,494	16,547,823,854	14.4	146,680,648	88.4	14,623,062,662	12.7
	KM04	273,704,658	27,437,151,474	23.9	254,536,358	25,448,112,544	22.1	223,447,485	87.8	22,339,899,850	19.4
	KM05	168,350,850	16,733,093,450	14.6	152,142,576	15,041,077,078	13.1	132,965,974	87.4	13,145,245,179	11.4
	KM06	187,680,176	18,955,697,776	16.5	164,290,448	16,481,695,975	14.3	140,528,756	85.5	14,097,911,719	12.3
	KM07	148,741,746	14,759,591,916	12.8	133,428,333	13,184,979,686	11.5	116,979,701	87.7	11,559,576,191	10.1
	KM08	146,955,038	14,668,017,348	12.8	131,815,747	13,103,215,080	11.4	115,891,661	87.9	11,520,272,764	10.0
	KM09	160,058,026	16,040,451,266	13.9	144,130,313	14,390,895,830	12.5	125,581,574	87.1	12,538,870,637	10.9
	KM10	204,214,514	20,625,665,914	17.9	182,640,777	18,353,461,016	16.0	157,919,889	86.5	15,869,273,960	13.8
	KM11	149,597,042	15,014,923,022	13.1	134,932,571	13,469,951,736	11.7	116,341,484	86.2	11,614,054,062	10.1
	KM12	192,884,606	19,334,384,986	16.8	171,250,612	17,101,998,930	14.9	150,667,904	88.0	15,046,499,997	13.1
	KM13	180,578,944	18,066,174,774	15.7	162,673,848	16,224,202,220	14.1	141,778,050	87.2	14,140,169,313	12.3
	KM14	165,943,924	16,672,030,554	14.5	149,183,948	14,945,605,966	13.0	130,801,337	87.7	13,103,991,876	11.4
	KM15	164,841,208	16,493,656,928	14.3	147,384,063	14,696,561,836	12.8	127,694,166	86.6	12,733,162,382	11.1
	KM16	183,096,344	18,387,552,612	16.0	170,223,481	17,057,565,402	14.8	150,423,946	88.4	15,073,515,603	13.1
Tama River estuary	TM01	167,227,406	16,684,654,744	14.5	149,314,565	14,868,396,395	12.9	129,195,977	86.5	12,865,034,290	11.2
Tsuchi River	TC01	149,706,638	15,021,817,278	13.1	136,790,787	13,679,441,455	11.9	118,787,975	86.8	11,879,112,513	10.3
	TC02	177,141,656	17,798,407,856	15.5	161,082,232	16,132,461,560	14.0	139,438,594	86.6	13,964,840,999	12.1
	TC03	163,548,752	16,426,050,538	14.3	154,675,701	15,499,482,777	13.5	136,095,685	88.0	13,637,647,750	11.9
	TC04	170,794,260	16,986,468,720	14.8	157,174,035	15,583,486,649	13.6	136,920,929	87.1	13,575,432,285	11.8
	TC05	149,683,016	14,999,963,036	13.0	134,031,405	13,373,334,493	11.6	115,530,242	86.2	11,527,332,496	10.0
	TC06	173,818,164	17,452,733,262	15.2	162,929,634	16,316,137,063	14.2	143,035,039	87.8	14,323,847,933	12.5
	TC07	169,555,642	17,125,119,842	14.9	151,133,441	15,225,149,411	13.2	130,998,805	86.7	13,196,790,635	11.5
	TC08	165,786,960	16,615,148,580	14.4	146,907,351	14,662,694,655	12.8	126,545,670	86.1	12,630,413,022	11.0
	TC09	171,977,990	17,369,776,990	15.1	155,530,889	15,646,687,897	13.6	134,744,936	86.6	13,555,583,543	11.8
	TC10	180,419,636	18,222,383,236	15.8	163,083,846	16,412,364,447	14.3	141,676,542	86.9	14,257,985,067	12.4
	TC11	139,489,716	14,011,410,796	12.2	122,621,626	12,276,732,777	10.7	106,502,345	86.9	10,662,889,348	9.3
	TC12	141,122,980	14,055,167,360	12.2	128,388,439	12,761,830,543	11.1	111,146,256	86.6	11,047,954,906	9.6
	TC13	180,002,980	17,690,189,328	15.4	163,108,603	16,008,092,873	13.9	141,972,909	87.0	13,933,756,227	12.1
Takase River	TK01	254,417,460	25,441,746,000	22.1	237,977,471	23,689,880,458	20.6	211,686,683	89.0	21,072,718,328	18.3
	TK02	251,669,932	24,975,015,718	21.7	243,356,024	24,123,799,421	21.0	214,306,633	88.1	21,244,143,227	18.5
	TK03	166,778,212	16,407,954,888	14.3	153,061,359	14,943,302,743	13.0	136,834,799	89.4	13,359,111,931	11.6
Mariana Ridge	MR01	111,119,606	11,111,960,600	9.7	107,283,045	10,715,949,315	9.3	93,488,061	87.1	9,338,039,606	8.1
	MR02	100,751,136	10,075,113,600	8.8	96,918,391	9,681,976,662	8.4	86,466,069	89.2	8,637,808,093	7.5
	MR03	145,533,522	14,553,352,200	12.7	138,626,007	13,847,300,788	12.0	123,297,729	88.9	12,316,164,743	10.7
	MR04	192,853,338	19,285,333,800	16.8	183,513,533	18,329,271,156	15.9	162,302,834	88.4	16,210,753,535	14.1
	MR05	128,008,122	12,800,812,200	11.1	122,894,913	12,276,066,067	10.7	108,208,537	88.0	10,809,032,830	9.4
	MR06	116,336,394	11,633,639,400	10.1	110,700,805	11,057,128,222	9.6	98,856,186	89.3	9,874,052,173	8.6
	MR07	165,375,126	16,537,512,600	14.4	158,627,732	15,846,015,023	13.8	142,316,884	89.7	14,216,653,377	12.4

Table S4. Comparison of mapping rates with eels of different species.

	Input reads	*Anguilla japonica*		*Anguilla anguilla*		*Anguilla rostrata*	
		Mapped reads	Mapped ratio	Mapped reads	Mapped ratio	Mapped reads	Mapped ratio
TW22	151,717,823	133,444,112	88.0	127,338,128	83.9	100,955,294	66.5
SG11	182,394,414	160,431,495	88.0	153,354,456	84.1	121,497,095	66.6
KM02	145,734,939	128,230,942	88.0	122,405,896	84.0	96,899,548	66.5
KM08	131,815,747	115,891,661	87.9	110,338,498	83.7	87,629,938	66.5
KM12	171,250,612	150,667,904	88.0	143,765,213	84.0	113,996,771	66.6
TC03	154,675,701	136,095,685	88.0	129,674,464	83.8	103,105,194	66.7
TK02	243,356,024	214,306,633	88.1	204,602,758	84.1	162,066,083	66.6
MR05	122,894,913	108,208,537	88.0	103,527,744	84.2	82,206,808	66.9
A. anguilla	67,974,339	59,372,788	87.3	63,317,574	93.1	49,979,926	73.5
A. rostrata	286,873,752	239,503,053	83.5	256,373,371	89.4	212,328,050	74.0

Table S5. Pairwise distance values among 30 individuals of Japanese eel *Anguilla japonica* based on 32,312,607 SNP sites.

	KM01	KM02	KM05	KM08	KM10	KM11	MR01	MR02	MR03	MR04	MR05	SG01	SG02	SG03	SG04	SG05	TC01	TC02	TC03	TC04	TC05	TK01	TK02	TK03	TM01	TW01	TW02	TW03	TW04	TW05
KM01	-																													
KM02	6354551	-																												
KM05	6010050	5739982	-																											
KM08	6197990	6443341	5841240	-																										
KM10	6185537	6486365	6312904	5985271	-																									
KM11	6366299	6511632	6282127	6468167	6401808	-																								
MR01	5911173	6422893	6063121	6084502	6318048	6259106	-																							
MR02	5914748	6411964	6066051	6084708	6313155	6243963	5458402	-																						
MR03	5904939	6400278	6055948	6071833	6297713	6232741	5462027	5465188	-																					
MR04	5901931	6395065	6057922	6070738	6292707	6223553	5472176	5463867	5466293	-																				
MR05	5915019	6414434	6069890	6090828	6313053	6244236	5471611	5477471	5472236	5479692	-																			
SG01	5905497	6395598	6050836	6075090	6289903	6228221	5462949	5474585	5468742	5479398	5483483	-																		
SG02	5899718	6393490	6051966	6066185	6292013	6225740	5470540	5474232	5468392	5472001	5480236	5471425	-																	
SG03	5897811	6391685	6049346	6070267	6290452	6223142	5464508	5465879	5459504	5467689	5471464	5467668	5462539	-																
SG04	5897121	6387069	6050070	6067800	6292624	6222248	5459211	5464632	5470060	5465933	5473834	5464429	5464194	5454879	-															
SG05	5897757	6386438	6050056	6066435	6288619	6219441	5463573	5470628	5466313	5467279	5477234	5463567	5465374	5465083	5460607	-														
TC01	5941360	6439230	6097654	6113254	6340165	6274388	5504739	5508999	5506435	5506053	5515906	5511257	5509220	5496764	5508320	5501273	-													
TC02	5980042	6442460	6131078	6137590	6342708	6277029	5572556	5578126	5568732	5571462	5582708	5571622	5567755	5563586	5563031	5564762	5192623	-												
TC03	5921677	6410761	6072150	6090027	6311741	6246853	5483996	5487632	5483473	5486195	5491855	5481815	5484422	5478461	5484446	5484843	5515355	5581038	-											
TC04	5939085	6421008	6087144	6109172	6318626	6253296	5513309	5525816	5518538	5515561	5524623	5512941	5514570	5507583	5510198	5512119	5547524	5579974	5391582	-										
TC05	5940869	6432318	6091565	6110481	6334042	6269080	5499045	5502892	5506227	5509410	5514683	5512849	5509450	5504065	5504946	5500847	5540765	5600752	5512814	5470783	-									
TK01	5894215	6383275	6044478	6062666	6280734	6214159	5462270	5465394	5464718	5463949	5476296	5465637	5468533	5460635	5456623	5466922	5503868	5568507	5486615	5514931	5502663	-								
TK02	5886526	6378685	6041953	6054935	6274973	6209346	5451173	5455841	5451097	5455943	5465269	5455001	5462103	5456917	5453614	5454206	5494298	5562039	5472831	5500646	5493491	5446330	-							
TK03	5900505	6391633	6050594	6064338	6292131	6224748	5463354	5469912	5466053	5467428	5472970	5463444	5468842	5460533	5457985	5461935	5504438	5570696	5480612	5511076	5505588	5456466	5445789	-						
TM01	5904856	6405536	6064257	6085100	6308520	6238382	5468730	5476742	5475024	5480261	5485602	5477021	5478534	5474251	5471044	5475529	5510224	5579744	5493640	5521421	5512323	5466970	5463575	5470803	-					
TW01	6104129	6599181	6253457	6269199	6494166	6432624	5650134	5665264	5658434	5662949	5670850	5659323	5661321	5656258	5656903	5660568	5698230	5765312	5679739	5706690	5703637	5659585	5647724	5656779	5667255	-				
TW02	5905335	6395401	6057686	6073558	6294100	6233846	5472371	5477638	5469588	5471939	5481528	5475406	5469917	5474465	5469919	5470881	5511708	5570112	5488406	5521426	5512455	5472303	5460153	5474936	5479127	5663234	-			
TW03	5922890	6418673	6076352	6091091	6316138	6250270	5484751	5493383	5483313	5487487	5496413	5490763	5490425	5476034	5485218	5490391	5525215	5588805	5506563	5534198	5528503	5486219	5473241	5479676	5497228	5671388	5485516	-		
TW04	5907629	6395369	6058617	6072499	6296802	6227740	5479254	5480040	5476149	5481862	5489134	5474618	5478886	5473246	5473541	5473423	5512847	5574689	5500612	5521954	5516010	5476765	5461964	5473316	5478674	5668349	5480401	5496881	-	
TW05	5916963	6410928	6067896	6079761	6302732	6240668	5470884	5476531	5479347	5482900	5490409	5474606	5479110	5475782	5473876	5475613	5512668	5577327	5495623	5523495	5513899	5478609	5465938	5471179	5481514	5655058	5480100	5492253	5482095	-

Table S6. The number of SNPs and the outlier SNPs site in each linkage groups.

Linkage group	SNPs	Outlier SNPs site		LOSITAN Outlier		
		BayeScan	LOSITAN	Ave. Fst	Min Fst	Max Fst
LG1	124,545	4	-	-	-	-
LG2	152,882	6	-	-	-	-
LG3	76,871	7	2,325	0.22	0.02	1
LG4	138,236	5	-	-	-	-
LG5	110,785	0	-	-	-	-
LG6	75,841	3	2,335	0.21	0.02	1
LG7	160,288	3	-	-	-	-
LG8	91,643	1	-	-	-	-
LG9	161,375	3	-	-	-	-
LG10	89,537	3	1,952	0.28	0.102	0.979
LG11	66,100	0	1,785	0.25	0.096	0.979
LG12	100,914	1	-	-	-	-
LG13	61,450	0	1,476	0.26	0.015	1
LG14	96,526	0	-	-	-	-
LG15	56,396	0	2,283	0.16	0.04	0.979
LG16	93,193	5	-	-	-	-
LG17	13,825	3	399	0.27	0.02	1
LG18	49,585	2	1,048	0.29	0.102	0.979
LG19	116,031	1	-	-	-	-

Table S7. Fixation index (Fst) values between each set of six regional populations of the Japanese eel *Anguilla japonica,* based on 32,312,607 SNP sites. This table corresponds to Figure 4.

	SG	TW	KM	TC	TK	MR
SG	-	0.000	0.014	0.001	0.000	0.001
TW		-	0.014	0.001	0.000	0.001
KM			-	0.014	0.011	0.013
TC				-	0.001	0.001
TK					-	-0.001
MR						-
